# Mapping how responsibility for poor diets is framed in the United Kingdom: a scoping review

**DOI:** 10.1017/S1368980025101079

**Published:** 2025-09-22

**Authors:** Nestor Serrano-Fuentes, Lyn Ellett, Christina Vogel, Janis Baird, Nuno Tavares, Mari Carmen Portillo

**Affiliations:** 1 National Institute for Health Research Applied Research Collaboration Wessex, Southampton Science Park, Innovation Centre, Southampton, UK; 2 School of Health Sciences, Highfield Campus, University of Southampton, Southampton, UK; 3 School of Psychology, Highfield Campus, University of Southampton, Southampton, UK; 4 Medical Research Council Lifecourse Epidemiology Centre, University of Southampton, Southampton General Hospital, Southampton, UK; 5 National Institute for Health Research Southampton Biomedical Research Centre, University of Southampton and University Hospital Southampton NHS Foundation Trust, Southampton, UK; 6 Centre for Food Policy, City University of London, London, UK; 7 Faculty of Science and Health, University of Portsmouth, Portsmouth, UK

**Keywords:** Diet-related diseases, Disadvantaged groups, Poor diet, Responsibility

## Abstract

**Objective:**

To identify and present (i) how responsibility for poor diets in the UK is framed across the public, mass media and the government and (ii) how groups experiencing socio-economic disadvantage are presented within this framing.

**Design:**

A scoping review of peer-reviewed literature was conducted using six databases. A systematic narrative synthesis guided by qualitative content analysis was applied to summarise the findings.

**Results:**

Thirty-six articles were included. Studies exploring public perceptions of poor diets acknowledged personal and broader systems drivers, with individual responsibility predominating across studies. Research analysing media portrayals showed similar patterns of individual responsibility among right-leaning newspapers, which focused on individual lifestyle changes. However, left-wing newspapers highlighted the role of the food industry and the government. Studies analysing government policies identified citizens as the primary agents of change through rational decision-making. Framing from socio-economically disadvantaged groups showed a preference for prioritising their own choice, but were limited by household income, food prices and family food preferences. Policies and media portrayals provided limited emphasis on these populations, with individual responsibility narratives prevailing.

**Conclusions:**

The framing of responsibility for poor diets in the UK centred on the individual, obscuring the powerful influence of food manufacturers and retailers and the role of government in providing safe, healthy environments for all. This review highlights the urgent need to challenge this narrative, with the public health nutrition community working collectively to force a radical shift in public, media and policy framing and incite strong regulatory action by governments.

The UK has diets with the highest proportion of ultra-processed food in Europe^(1)^. The population’s diet is also too high in carbohydrates, total fats, saturated fats, salt and free sugars and does not meet fibre, protein and potassium recommendations^(2)^. This suboptimal nutrition underlies an increase in diet-related conditions, such as obesity, type 2 diabetes, CVD and several types of cancer, and the financial costs attached to them^(2)^. In the UK, the combined cost of obesity and associated health issues in reducing life expectancy, National Health Service funds and lost workforce productivity is £98 billion yearly^(3)^.

A major cause of poor diets is the influence of the obesogenic food environment^(4)^. This term describes settings in which unhealthy food is widely accessible, available and affordable, creating conditions that drive people to make unhealthy food choices and fuelling obesity at a population level^(5)^. These obesogenic environments are largely driven by the power and influence of the food industry, which prioritises producing and promoting ultra-processed food^(2)^. As a result, people’s dietary patterns are shaped in ways that operate beyond individual awareness and control and embed unhealthy foods as the social norm within everyday social practices and routines^(6)^. The lower pricing and targeted marketing of unhealthy food products are particularly concentrated in disadvantaged communities. This systematic targeting, combined with financial constraints that limit the food choices of families living on lower incomes, perpetuates dietary and health inequalities^(7)^.

Evidence shows that government-enforced regulatory approaches like the UK Soft Drinks Industry Levy (2018)^(8)^ and legislation restricting the promotions of high in fat, sugar and salt products in prominent locations of retail outlets^(9)^ effectively improve obesogenic food environments by creating a level playing field for the food industry. In contrast, industry-led voluntary or corporate social responsibility initiatives like the Public Health Responsibility Deal (2011–2015) have proven ineffective. Independent evaluations show that companies word their pledges vaguely to enable poor quality progress reports and dodge robust monitoring or prioritise only the easiest health targets, which do not negatively impact their business strategies^(10)^.

Despite this evidence base, comprehensive government-enforced regulatory interventions to reshape obesogenic food environments remain limited in the UK, with recent proposals for stricter rules repeatedly delayed or discarded^(11)^. This is because implementing these interventions faces significant barriers, as the food industry acts to protect commercial interests throughout the policy process^(12–16)^. Research investigating the involvement of alcohol and tobacco industries in public health policy making shows these industries systematically exercise structural power through argument-based and action-based strategies^(13)^. It is important to investigate whether these strategies are also used by the food industry. Argument-based strategies include framing health issues as matters of individual choice (e.g. tobacco companies promoting ‘freedom to choose’), positioning as legitimate stakeholders (e.g. alcohol industry being ‘part of the solution’) and highlighting economic benefits (e.g. job creation). Action-based strategies involve building coalitions against regulations (e.g. tobacco companies partnering with hospitality associations against smoke-free laws), funding favourable research (e.g. Coca-Cola sponsoring physical activity studies and shifting focus from their products’ role in obesity to sedentary behaviours)^(17)^, securing positions on regulatory committees (e.g. a legislator becoming a Tobacco Institute lobbyist^(18)^, creating legal obstacles to implementation and intimidating public health advocates^(15)^.

While many working in the field of public health are calling for stronger government regulation of the food industry, this effort faces significant barriers without greater public support for these policies^(19)^. Public support is key because it is one of several factors policymakers consider before implementing a policy, alongside the policy’s likely costs and effectiveness^(20,21)^. Public support may be limited by a critical disconnect. While research evidence demonstrates that environmental and commercial factors are primary drivers of poor diets, many citizens may largely perceive that individuals are responsible for their food choices^(22,23)^.

Understanding this disconnect requires examining how responsibility is framed across the key domains that shape public understanding in their everyday lives: public discourse, mass media and government policy^(24,25)^. Research shows that societal framing (e.g. industry manipulation or toxic food environment) in health news articles increases policy support^(26–28)^, while individual-focused frames (e.g. in obesity) decrease support for government intervention^(27,29)^.

However, there is limited comprehensive evidence mapping how responsibility for poor diets is currently framed across these influential domains in the UK. While previous studies have examined responsibility framing in single domains^(30,31)^, a comprehensive synthesis is needed to understand the complete picture of responsibility narratives that citizens encounter. Furthermore, how disadvantaged groups – who bear a disproportionate burden of diet-related ill-health^(32)^ – are represented within responsibility framing has received limited systematic examination^(28)^. If responsibility framing fails to acknowledge societal barriers and instead emphasises individual choice, it may perpetuate stigma and reduce support for policies that could address the root causes of dietary inequalities.

This study aims to address these evidence gaps by conducting the first systematic synthesis of how responsibility for poor diets is framed across public perceptions, mass media and government policy in the UK, with particular attention to the representation of socio-economically disadvantaged groups.

## Methods

A scoping review with systematic methodology was selected as it allows for rigorous exploration of a broad review question, mapping and summarising the breadth and depth of available evidence across multiple time periods and domains (the public, mass media and the government). This approach enables the identification of patterns and knowledge gaps that would not be captured through primary analysis of contemporary sources alone, thereby informing future research^(33,34)^. Assessment of the methodological quality of the included studies was not completed^(35)^.

The review followed the Joanna Briggs Institute methodology for scoping reviews^(36)^ and reported following the Preferred Reporting Items for Systematic Reviews and Meta-Analysis extension for scoping reviews (PRISMA-ScR)^(37)^.

This method was supported by framing theory. Framing refers to the process by which certain aspects of an issue are highlighted, while others are left out, encouraging audiences to think, feel and decide in a particular way^(38)^. We specifically focused on responsibility frames – those that attribute causation and solutions for health problems (in this case, poor diets) to different actors or levels, ranging from individuals and social relationships to broader societal structures^(39,40)^.

### Review questions

The review questions were: (a) ‘What framing has been used among the public, mass media and government about who is responsible for poor diets in the UK?’; (b) ‘How are disadvantaged groups presented within this framing?’ The questions were developed using the Participants, Concept and Context framework (see online supplementary material, Supplemental Material 1 for detailed definitions of the key terms and components of the review questions).

### Search strategy

A full search strategy (see online supplementary material, Supplemental Material 2) was developed utilising search terms from the titles and abstracts (keywords and/or medical subject headings or subject headings) related to food environments, commercial and policy influences, diet and nutrition, media and digital platforms, framing constructs related to responsibility and the UK and combined with the Boolean terms ‘OR’ and ‘AND’. The final search terms were based on previous literature, team discussions and a librarian’s input. The search strategy, including all identified index terms and keywords, was adapted for each database and information source.

The databases included MEDLINE (Ovid), CINAHL (EBSCOhost), PsycINFO (EBSCOhost), Web of Science, EconLit (EBSCOhost) and GEOBASE, covering health, geographic marketing/economic and consumer literature. The reference lists of included literature were screened for further sources. The search strategy limited the publication date to include sources from the year 2000, when literature on environmental influences on diet-related conditions (above all, obesity) emerged. Only English-language literature was included due to the UK focus. The original search strategy was updated twice (November and December 2023) to incorporate terms related to the ‘online food environment’ and then ‘policy’ and ‘news’. These modifications ensured coverage of public, mass media and government framing on who is responsible for food choices and poor diet in the UK. While our search strategy was designed to capture mass media broadly, the identified studies examining media framing of responsibility for poor diets were limited to newspaper coverage, reflecting the current state of the literature in this domain.

### Study selection

**Figure 1.**
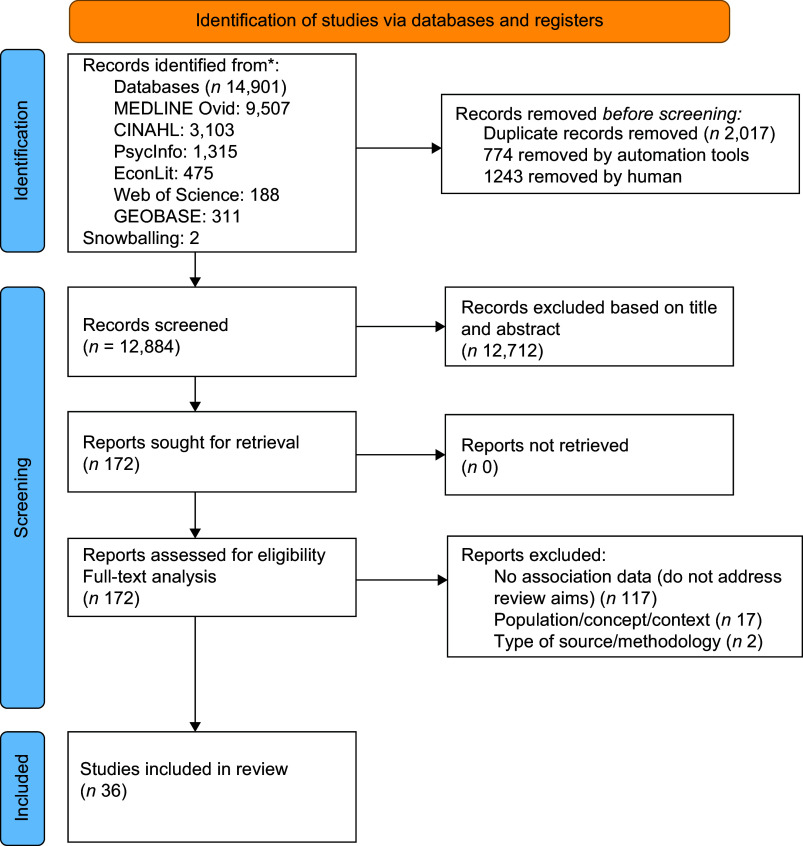
PRISMA flow diagram.

All identified records were uploaded into EndNote v.20, and duplicates were removed automatically and double-checked (and removed if necessary) manually. NSF assessed titles and abstracts. NSF and NT independently screened the full texts of the selected articles for eligibility, with a percentage agreement at 98·8 %. Discrepancies were resolved through discussion. The inclusion and exclusion criteria are specified in Table 1. The identification process and the search results are presented in a PRISMA flow diagram^(41)^ (Figure 1).

**Table 1.** Inclusion and exclusion criteria

Inclusion criteria	Exclusion criteria
Articles published in peer-reviewed journals: Primary inclusion: studies directly examining responsibility attribution for poor diets or diet-related conditions in the UK Secondary inclusion: studies investigating factors influencing food choices, where responsibility attribution could be inferred from the framing of these factors	Intervention studies to increase awareness of the power and influence of the food industry and government on diet practices/diet-related conditions
Exploratory research (non-experimental quantitative, qualitative study designs (including critical analysis) and mixed-method designs)	Articles that did not meet the Participants, Concept and Context criteria for the study
Literature reviews	Inaccessible full texts
	Grey literature (e.g. conference abstracts or student theses and dissertations)

### Data extraction and analysis

Data were extracted utilising a data extraction tool (see Table 2) developed for this scoping review^(36)^. Data extraction captured information about the author[s], year of publication, study aim, type of evidence and study design, study population and context and key findings on responsibility for poor diets. NSF extracted 100 % of the data. Charting the results was iterative, allowing for emergent data throughout the data extraction process and revising and modifying the data extraction tool as necessary. Disagreements were resolved with the rest of the authors (LE, NT, MCP, JB and CV).

**Table 2.** Extraction table

Selected studies (author, year)	Aim	Type of evidence and research method	Population and/or resources studied	Key findings
Backett-Milburn *et al.*, 2006^(60)^	To examine parents’ views about their teenagers’ tastes and eating behaviours and consider the meanings attached to eating at home and away from home	- Qualitative data - Iterative qualitative approach using interviews	- Thirty-four parents of teenagers aged 13–14 years living in socio-economically disadvantaged circumstances	Factors influencing eating practices: - Individuals (parents or teenagers) (main responsible). - Individual responsibility influenced by family budget and work/leisure commitments - Awareness of junk food around
Barker *et al.,* 2008^(61)^	To identify factors that influence the food choices of women with lower educational attainment and how women could be helped to improve those choices	- Qualitative data - Focus groups analysed through coding reliability	- Forty-two adult women aged 18–44 years (the majority with lower educational attainment)	Food choices are influenced by: - Perceived lack of control over food choices (the most important) - Partners and children’s preferences - Higher prices of healthy food - Looking after children - Historical family influences
Beeken and Wardle, 2013^(62)^	To assess attributions for overweight and the level of support for policy initiatives in Great Britain	- Quantitative data - Cross-sectional study, online survey analysed through descriptive statistics	- 1986 adults (mixed sociodemographic characteristics)	Causes of obesity (from more to less important): - Too many unhealthy foods around - Person’s own fault - Lack of willpower - Genes
Chambers and Traill, 2011^(63)^	To study what the UK public believes to be the causes of obesity and the relationship between these beliefs and support for potential policy interventions	- Quantitative data - Cross-sectional, survey, analysis through principal component analyses using Varimax rotation	- 500 adults (≥ 18 years old) with different sociodemographic characteristics	Causes of obesity: - Unhealthy foods too readily available - Individual responsibility (lack of willpower to diet and exercise) - Genes, the least important cause
Cook *et al.*, 2021^(64)^	To uncover the barriers and facilitators that help or hinder parents’ ability to provide a healthy diet to prevent overweight and obesity among their young children	- Qualitative data - Interpretative qualitative study with a phenomenological perspective, focus groups analysed through a framing approach	- 110 parents of children aged 0–5 years from deprived and ethnically diverse wards	Factors influencing diet practices: - Mother, responsible for the household diet - Mothers influenced by lack of time, work and childcare, affordability of healthy food, exposure to unhealthy food, past childhood experiences, other family members - Parents, role models for their children
Crawshaw and Newlove, 2011^(50)^	To consider men’s responses to social marketing strategies and their own understandings of health, its determinants and personal responsibility	- Qualitative data - Semi-structured focus groups and individual interviews, analysed through thematic analysis	- Fifty unemployed men aged 20–55 years	Factors influencing health: - The individual (main responsible) - Price of healthy food - Time constraints - Family influences - Limited income
Devi *et al.,* 2010^(65)^	To explore the factors influencing schools’ decisions and children’s food choices in relation to vending machines	- Qualitative data - Semi-structured interviews and focus groups	- Thirty-one school staff and students	Factors influencing food choices: - The importance of having personal choice and freedom (individual responsibility) - School and family responsibility - Students frustrated with the government for imposing ‘unfair’ and ‘harsh’ policies on their freedom
Dhuria *et al.,* 2021^(22)^	To examine women’s perceptions of factors that influence their food shopping choices, particularly in relation to store layout, and their views on ways that supermarkets could support healthier choices	- Qualitative data - Qualitative cross-sectional study with semi-structured interviews, analysis through thematic analysis	- Twenty women aged 18–45 years, most of them living in deprived neighbourhoods	Factors influencing food choices: -Personal responsibility (main factor): self-control, better organisation and plannification for eating practices Factors that influence the individual: - Accessibility - Time - Work - Family influence - Product placement strategies - Food prices - Mood or general state of mind
Dibsdall *et al.,* 2002^(66)^	To provide an in-depth account of the beliefs and experiences pertaining to food and health from a specific group of low-income women in the UK	- Qualitative data - Semi-structured interviews, analysis through the interpretative phenomenological method	- Fourteen women aged 40–60 years from a defined low-income group	Factors influencing food choices (no factor is more important than another): - Individual responsibility - Historical family reasons - Time constraints - Price of healthy food
Douglas *et al.,* 2014^(67)^	To explore mothers’ perspectives about the nature and causes of childhood obesity, their views and experiences of managing their child’s weight and about effective weight management strategies for this age group	- Qualitative data - Grounded theory, focus groups, thematic analysis	- Thirty-four mothers (aged 23–42 years from a range of socio-economic backgrounds) of children aged 3–4 years	Causes of childhood obesity: - Parental failure (the most important) - Structural factors: historical family influences, the cost of food, reduced time available, widespread availability of unhealthy food, food marketing strategies - Family influence
Goldthorpe *et al.*, 2019^(51)^	To explore children’s views about who they feel is responsible for keeping them healthy	- Qualitative data - Focus groups, interpretative phenomenological analysis	- Twenty children aged 8–10 years old, from primary schools in deprived inner city areas	Responsibility for being healthy: - Individual and environment (no differences) - Individual (preventative self-care) - The government should provide more money for public health initiatives - Accessibility, convenience and relatively low price of fast food influence individual’s decision-making - Schools - Parents - Food business marketing
Greener *et al.*, 2010^(68)^	To understand the causes of obesity/overweight, beliefs about factors that enabled or inhibited weight loss/gain and opinions regarding effective obesity/overweight interventions	- Qualitative data - In-depth individual interviews analysed with a framework approach	- Sixty-three adults (lay self-identified overweight adults aged 18–50 years, health professionals and policymakers)	Causes of obesity: - Personal factors (e.g. lacking motivation) - Genes - Family influence (poor role modelling) - Illness and disruptive life events
Gregg *et al.*, 2017^(52)^	To present a novel research design method, netnography, by utilising it to summarise in real-time, the public’s reactions to the publication of the childhood obesity strategy with the purpose of informing subsequent policy, practice and government action	- Qualitative data - A netnographic technique of reviewing user-generated online content	- Three newspaper articles and 1704 associated comments related to the policy on *Childhood obesity: a plan for action*	Responsibility for childhood obesity: - Parents (ultimately responsible) - Wide acknowledgement of government responsibility - Solutions around nutritional education and the cost of healthy food and changing societal norms - The influence of the food industry; supermarkets had an opportunity to have an impact but are not engaging in public health initiatives
Hardcastle and Blake, 2016^(69)^	The purpose of this qualitative study was to explore the perceptions and attitudes that underlie food choices and the impact of a school-based healthy eating intervention in mothers from an economically disadvantaged community	- Qualitative data - Semi-structured interviews, thematic content analysis	- Sixteen mothers from a socially deprived community	Factors that influence food choices: - Food price - Parents - Socialisation - Historical family reasons
Harden and Dickson, 2014^(70)^	To enhance understanding of the wider contexts within which family food practices are developed, this study examined the experiences of low-income mothers with young children	- Qualitative data - Qualitative longitudinal design, individual interviews, inductive thematic analysis	- Thirteen mothers (aged 18–40 living in socio-economically deprived areas) of children 6 years and under	Factors that influence food choices: - Individual responsibility (the main cause) - Not having enough money - Price of healthy food - Family influences
Hayter *et al.*, 2015^(71)^	To explore parental perceptions of feeding their children in order to inform the development of a nutrition intervention	- Qualitative data - Focus groups and individual interviews informed by framework analysis	- Thirty-nine parents of children aged 18–39 months from deprived areas	Factors that influence food choices and purchases (no factor is more important than another): - Not having enough money - Price of healthy food - Time constraints - Family influences - Food marketing strategies - Cooking skills - Historical family reasons - Parents, role models - Peer influence
Khanom *et al.*, 2015^(72)^	To elicit evidence on the main barriers and facilitators to dietary choice and to inform the development of interventions that they would like to see put in place to promote a healthier food environment for their children	- Qualitative data - Inductive qualitative research with semi-structured interviews	- Sixty-one parents aged 20–52 years living in deprived areas	Factors that determine infants’ diets: - Community and culture - Financial barriers (major reason) - Shift work - Caring for children - Activities outside the home - Limited access to personal transport (accessibility), no local shops within a local distance - Lacked cooking skills - Family influence - Food marketing strategies in supermarkets - Solutions: more healthy foods in supermarkets; reduce promotions on unhealthy foods; the government should ensure that food manufacturers produce food low in salt, sugar and fat content and subsidise cheaper, healthier food; ‘fast food’ outlets restricted; more access to local, healthy food
Lawrence *et al.*, 2009^(73)^	To provide insight into factors that influence the food choices of women with lower educational attainment	- Qualitative data - Focus groups, thematic analysis	- Fifty-six women aged 18–44 years of lower educational attainment	Factors that influence food practices (no factor is more important than another): - Food costs - Food marketing strategies - Time pressure - Family influences - Historical family factors - Self-efficacy, lack of confidence in cooking skills
Ludwig *et al.*, 2011^(74)^	To explore health perceptions, diet and the social construction of obesity and how this relates to the initiation and maintenance of a healthier diet in UK Pakistani women	- Qualitative data - Focus groups and individual semi-structured interviews, analysis through phenomenological and sociological approaches	- Fifty-five Pakistani women aged 23–80 years	Causes of obesity: - Individual responsibility: laziness to exercise or change eating habits, lack of concern for preventing illness - Family influences on cooking choices - Weight gain as natural and unavoidable (childbirth and age) - Climate - Social norms: the social importance of cooking for guests and of celebratory meals
Muir *et al.*, 2023^(9)^	To (i) assess stakeholders’ views on the legislation, including their perceived benefits, concerns and support needs arising from its implementation, using a pre-implementation rapid qualitative evaluation, and (ii) determine and prioritise recommendations for policy, using participatory techniques	- Qualitative data - A pre-implementation rapid qualitative evaluation	- 108 consumers, businesses, enforcers and environmental health officers	Responsibility for poor diets: - Environment (main responsible) - Government plays a vital role
O’Brien *et al.*, 2015^(75)^	To examine the role of health in consumers’ food purchasing decisions through investigating the nature of people’s discourse regarding health while conducting their food shopping	- Qualitative data - Think-aloud technique as part of an accompanied shop, inductive thematic analysis	- Fifty adults with varied sociodemographic characteristics	Factors influencing food choices: - Individual responsibility (e.g. lack of self-control) (main cause) Individual responsibility is affected by: - Family influences - Time constraints - Price of foods
Swift *et al.*, 2018^(76)^	To explore UK public perceptions of children’s sugar consumption, Public Health England’s Change4Life Sugar Smart app and the Soft Drinks Industry Levy, using solicited and unsolicited digital data	- Mixed-methods data - Online questionnaire; posts to UK online parenting forums and English language Tweets from Twitter. Quantitative data were analysed using descriptive statistics and qualitative data using content and inductive thematic analysis	- 184 participants, 412 forum posts, 618 Tweets	Barriers to reducing the amount of sugar that children have (from most important to less important): - Environments that encourage consumption - Media and advertising - Parents’ lack of willingness to change their own food/drink choices - Lack of knowledge about selecting low-sugar alternatives - Children’s taste preferences - Lack of suitable alternatives - Higher cost of low-sugar alternatives - Extra meal planning to incorporate low-sugar alternatives
Thomas-Meyer *et al.*, 2017^(53)^	To capture the views, ideas and concerns of commenters on major UK news websites on sugar-sweetened beverages taxes	- Qualitative data - Qualitative analysis of reader comments on online news coverage	- 1645 comments on four articles	Responsibility for food and drink choices: -Individuals (main responsible) through overconsumption and lack of physical activity, no responsibility on society or government
Timotijevic *et al.*, 2018^(54)^	To explore in depth how young people conceptualise personal responsibility vis-à-vis childhood obesity, in relation to their own bodies and in terms of societal and collective health. It will also examine the extent to which they understand childhood obesity in terms of societal, as opposed to individual, responsibility	- Qualitative data - Focus groups, inductive analysis	- Eighty-one adolescents aged 13–18 years	Responsibility for obesity: - Individual - Families - Schools - Price of food - Influence of peers
Watts *et al.*, 2023^(77)^	To understand factors that influence food choice and explore public perceptions of the need for government policies to improve diets in the UK, particularly food pricing interventions	- Qualitative data - Qualitative study design with semi-structured interviews informed by framework analysis	- Fifteen adults from a diverse range of backgrounds	Factors that influence food choice (no one is better than other): - Price of food - Food marketing strategies - Time constraints, work and family commitments - Eating culture - Lack of knowledge or confidence - Willpower to eat healthily - Government intervention is unfair; responsibility on consumers
Atanasova and Koteyko, 2017^(55)^	To explore obesity frames and their frequency of use in British and German online newspapers	- Mixed-methods data - Content analysis of news using inductive and deductive phases	- Mass media (press)	Causes of obesity: - Self-control (most important) - Environment (2nd most important): living environments and availability/affordability of foods - Nutritional education (3rd most important)
Baker *et al.*, 2020^(30)^	To examine the ways in which obesity has been framed by the press over a ten-year period (2008–2017), focusing both on areas of stability and change	- Mixed-methods data - Content analysis of news using corpus linguistics	- Mass media (press)	Causes of obesity: -Food manufacturers have taken up less space in debates around obesity over time - Obesity has been discussed less as a political issue. - Food intake tends to be framed as people making healthy eating choices as opposed to the regulation of food marketing strategies
Brookes and Baker, 2022^(56)^	To examine how the UK print media represents risk in reporting about obesity	-Mixed-method data - Corpus linguistic methods combined with qualitative discourse analysis of news	- Mass media (press)	Responsibility for obesity: - *The Guardian* openly criticises the government for an insufficient response to obesity; *The Independent* is less critical - Left-wing newspapers focus on food manufacturers, the government and health authorities. Right-wing newspapers focus on modifying personal behaviours - Increasing individual responsibility over time, obesity-related to personal choice (74 % of cases), as opposed to biological factors (18 %) or socio-political factors (8 %)
Busam and Solomon-Moore, 2023^(48)^	To examine how childhood obesity is framed by news articles on Facebook and how individuals commenting understand and react to these articles	- Qualitative data - Experiential qualitative design exploring textual data analysed deductively using framing analysis	- Mass media (press) and comments from the news	Causes of childhood obesity (in the news): - Societal factors (most prevalent), followed by behavioural and medical Public voices of causes of obesity (in the comments) (do not mention which cause was more important): - Individual’s diet and physical activity behaviour - Parents influenced by working conditions, salary and lack of time - Schools - Societal influences (policy, financial pressures and the food industry)
Elliott-Green *et al.*, 2016^(57)^	To assess the extent of media-based public health advocacy *v*. pro-industry messaging regarding sugar-sweetened beverages	- Mixed-method data - Systematic analysis of news articles using a coding framework and contextual analysis	- 374 articles from 25 national newspapers	Responsibility for consumption of sugar-sweetened beverages: - 14 % of British press articles negatively depicted the food industry’s role in promoting sugar, with most portraying the industry as neutral - Online newspapers emphasised individual responsibility more than print newspapers (12 % *v*. 9 %) - 24 % of articles proposed policy solutions, 31 % placed responsibility on individuals for reducing sugar consumption. - Broadsheets more likely than tabloids to suggest policy changes in addition to individual responsibility
Hilton *et al.*, 2012^(31)^	To examine the evolution and framing of the obesity epidemic over the past 15 years in British newspapers to identify any shifts in news coverage about the causal drivers of, and potential solutions to, the obesity epidemic	- Mixed-methods data - Thematic content of articles	- Seven UK newspapers and 2414 articles	Causes of obesity: - Individual drivers (the most common) (e.g. lack of exercise). - Mid-market articles emphasised individual drivers more, while serious articles highlighted societal drivers more (e.g. food advertising) - Articles mentioning individual obesity drivers declined from 2001 to 2010
Attree, 2006^(49)^	To examine contemporary public health policies aimed at improving diet and nutrition, identifying the underlying theories about the influences on healthy eating in poor families and exploring the extent to which these assumptions are based on experiential accounts	- Systematic review with meta-synthesis	- Public health policies in relation to diet and nutrition in low-income households *Saving Lives: Our Healthier Nation (1999), Tackling Health Inequalities: A Programme for Action (2003), Choosing Health (2004), Choosing Health: Choosing a Better Diet (2004)*, *Healthy Start (2004)*	Responsibility for diet and nutrition: - Prime responsibility with the public, not with the NHS or the government - UK government to increase awareness of healthy eating; currently, it is not considering the psychosocial and cultural aspects of food consumption
Brookes, 2021^(58)^	To present a critical discourse analysis of the *Tackling Obesity: Empower People to Make Healthier Choices* Policy	- Qualitative data - Qualitative critical discourse analysis	- Policy *Tackling Obesity: Empower People to Make Healthier Choices*	Responsibility for obesity: - Individuals’ lifestyle choices (lack of knowledge) - Parents - Food business is presented positively - Government: minimal level of commitment
Griffin *et al.*, 2021^(78)^	To analyse *Childhood obesity: a plan for action* using a social determinants of health perspective	- Qualitative data - A realist approach with an analysis of policy discourses	- Policy: *Childhood obesity: a plan for action*	Causes of childhood obesity: - Focus on personal choice and behaviour change (particularly of parents) - Making healthier choices easier by providing nutritional information
Piggin, 2012^(79)^	To trace the development and production of a major UK social marketing campaign named Change4Life and examine how ideas about the causes of and solutions to the obesity epidemic are produced in differing ways throughout the health promotion process	- Qualitative data - Policy archaeology and semiotic analysis	- Campaign *Change4Life*	Causes of obesity: - Individuals victims of an obesogenic environment - Parents as active agents in explanations of health behaviours
Ulijaszek and McLennan, 2016^(59)^	To examine the shifting of the framing of obesity in UK policy in the years preceding Foresight Obesity, then post-Foresight to 2015	- Qualitative data - Textual policy analysis	- Twenty-two policy documents by the UK government (framing obesity)	Responsibility for obesity: - Individual (main responsible) - Power dynamics in obesity governance processes have remained unchallenged by the UK government

A narrative synthesis of findings through basic deductive-inductive qualitative content analysis complemented the tabular data^(42)^. Content analysis was chosen for its descriptive nature and applicability across study designs^(42)^. A deductive-inductive approach (deductive first) was selected to synthesise knowledge without prior assumptions^(43)^. Initial coding was based on the review questions and an analytical framework developed, informed by multilevel health determinants theory^(44–46)^, distinguishing between micro-level, community-level and structural-level factors. This helped systematically categorise the diverse range of influences on food choices identified across studies (see online supplementary material, Supplemental Material 1). Absolute frequencies of outcome types in the literature were used to aid the narrative synthesis of the findings and as a reflection of the current literature available about who is responsible for poor diets in the UK.

The analysis consisted of three phases^(47)^, and a detailed explanation of how categories, codes and the narrative were created can be seen in online supplementary material, Supplemental Material 3. The phases were (a) preparation (immersing in the data and becoming familiarised with the content), (b) organising (an initial unconstrained framework was used to extract data and modified inductively to address the review questions effectively) and (c) reporting (weaving together the analytic narrative and data extracts).

It is relevant to note that, as is common in scoping reviews, this review included studies with diverse research focuses. Studies were included if they directly examined responsibility attribution for poor diets and if they investigated factors influencing food choices in ways that allowed for interpretation of implied responsibility attributions. For studies in the latter category, we applied the deductive-inductive approach to extract information about implied responsibility. We analysed how factors like food prices or marketing were framed regarding individual agency, interpreting external constraints (e.g. high cost of healthy food) as indicating societal responsibility and personally actionable factors (e.g. motivation) as individual responsibility.

## Results

A total of 14 901 records were identified, 14 899 through database searches and two from reference lists. After removing duplicates, 12 884 remained for preselection, of which 12 712 were excluded based on the title and abstract. After applying the inclusion/exclusion criteria, 172 articles were considered for full-text analysis. Of these, 117 articles did not address the review aims, seventeen studied other types of populations and considered other contexts and two did not meet the study type criteria. Thirty-six articles (twenty-seven qualitative, two quantitative, six mixed-methods and one review with meta-synthesis) were included in the final analysis. Figure 1 outlines the flow of studies through the inclusion process, and Table 2 describes the included studies.

Of the thirty-six included studies, twenty-seven explored public perceptions of responsibility and drivers of poor diets; six examined news content related to poor diets and diet-related conditions, especially obesity; and five studied the frames characterising UK government initiatives to address poor diets and diet-related conditions. Two studies^(48,49)^ covered multiple topics and therefore were coded against all relevant themes described below. Also, eleven studies^(49–59)^ directly investigated responsibility attributions, while fifteen studies^(9,22,30,31,48,60–79)^ examined factors influencing food practices or causes of obesity, from which responsibility attributions could only be inferred.

### Framing of responsibility in public perceptions

Twenty-seven studies referred to public perceptions of poor diets and potentially associated conditions like obesity. Thirteen studies identified personal factors as the key determinants, suggesting individual responsibility attributions^(22,49,50,52,53,60,61,63,64,67,68,70,75)^, such as perceived lack of control over food choices^(53,61,73)^, willpower^(62,63,68,77)^, self-care^(80)^ and mood when shopping^(22)^. The influence of parents was identified as part of individual responsibility, specifically their decision-making on food purchases and eating choices, and as role models in healthy eating^(22,48,52,64,65,67,71,80)^, food preparation (not cooking from base ingredients) and provision of ready-made meals^(67,72,73)^ and giving in to children’s requests for unhealthy foods^(60,69)^.

Three articles studying public perceptions of poor diet identified individual and external factors as the most important in poor diets^(9,62,76)^. These articles showed that citizens viewed responsibility as stemming from food environments providing greater affordability, accessibility and visibility of unhealthy food rather than personal choice^(9,62,76)^. Eleven articles acknowledged both individual and environmental responsibility without prioritising one over the other^(48,54,65,66,69,71–74,77,80)^.

A range of external factors were identified as influencing eating practices (e.g. shopping, cooking and eating) beyond individual control, suggesting societal responsibility attributions. These included financial constraints, like living on a restricted family budget that constrained healthy food purchases^(48–50,70–72)^, and high costs of healthier food like vegetables and fruits (on many occasions, families would like to buy these products, but felt they could not afford them), compared to energy-dense processed food^(22,50,52,54,60,61,64,66,67,69–71,73,75,77)^. Limited time due to, for example, childcare^(61,64,72,73)^ and work schedules^(22,48,60,64,72,77)^ influenced families’ ability to undertake home cooking and consume a better diet quality^(22,48,50,64,66,67,71,73,75,77)^. Furthermore, cultural norms reinforcing unhealthy eating patterns^(72,74,77)^, unhealthy food industry marketing tactics^(48,50,67,72,80)^ like product placement and promotions on unhealthy food^(22,71,73)^ and limited government support to individuals and schools to buy healthier food were cited^(49,52,52,80)^.

As part of the inferred societal responsibility attribution, the community environment also influenced the diet practices of individuals. For example, schools provided nutrition education and skills to support healthy food choices^(48,54,65,80)^, and children interacted with friends and imitated their eating practices^(54,69,71)^. Poor access to stores with healthier food options (e.g. no local shops within walking distance or lack of access to transport) led to reliance on takeaway foods^(22,72)^, and a high exposure to unhealthy food due to a saturation of takeaway restaurants and convenience stores in the local areas^(63,64,67,80)^ was also associated with poorer diets.

Individual factors involved family (e.g. partners and children) pressures for purchasing unhealthy foods, which were preferred by family members, yet not on the shopping list^(22,50,61,64,67,68,70–75)^. Members of the family spoiled children with unhealthy treats like sweets and chocolate, regardless of what the children were allowed at home^(54,64,67,70,71)^. Other studies highlighted historical family influences on diets, mainly individuals who had not been taught to cook or experienced a limited range of foods from childhood showed poorer dietary practices later in life^(61,64,66,67,69,71,73)^.

Of studies examining perceptions among specific groups, fourteen focused on groups experiencing disadvantage^(22,49,50,60,61,64,66,69–74,80)^, five included samples with a range of sociodemographic characteristics^(62,63,67,75,77)^ and eight did not specify any indices of deprivation or socio-economic status^(9,48,52–54,65,68,76)^. Among the studies involving groups experiencing disadvantage, seven identified personal factors as the most important^(22,49,50,60,61,64,70)^. All articles targeting these groups identified income and food prices, time constraints and family influences as the main external factors influencing diet practices. Two of these studies examined comparisons between populations with different educational attainments^(61,73)^. Both articles indicated differences between groups, showing that adults with higher educational attainment held greater levels of control over food choices, received greater social support from their families for healthy eating (e.g. sharing food preferences for healthy eating) and were less constrained by environmental or contextual factors.

### Framing of responsibility in UK mass media (press)

The identified studies examining media framing of responsibility for poor diets were limited to newspaper coverage, as no studies examining other media formats were found. Six articles critically analysed UK newspapers’ narratives and discussions around who or what is responsible for poor diets and diet-related conditions. Specifically, one article explored the responsibility for sugar-sweetened beverages^(57)^. The remaining five articles investigated who held responsibility for obesity^(30,31,48,55,56)^.

The media largely portrayed poor diets and obesity as matters of individual responsibility^(30,31,55–57)^. In particular, individuals lacking self-control^(55)^ and knowledge about nutrition and food preparation^(55)^ and made poor food and dietary choices, such as dieting^(30,31)^ or consuming too much sugar^(57)^. Contextual determinants of poor diet received less focus as key causes of health problems, suggesting limited emphasis on societal responsibility. For instance, one study found that only 14 % of British press articles negatively depicted the food industry’s role in promoting sugar consumption^(65)^. Other factors covered in the media included poor food labelling, lack of nutrition education, unhealthy drink and food advertising and promotions and prevalence of fast-food outlets^(31,55)^. The work conducted by Busam and Solomon-Moore^(48)^, who analysed childhood obesity coverage in the media between 2015 and 2020, showed a single exception in the media framing. They identified environmental causes as the primary drivers covered (indicating a focus on societal responsibility), followed by individual lifestyles and biological and medical factors.

The framing of responsibility for poor diets and diet-related conditions differed by the type of newspaper. This finding is particularly important when considering different reader groups. Left-wing broadsheets (*The Guardian*, *The Independent*) and left-wing tabloids (*Mirror*) emphasised the role of the government, food industry and health authorities more than right-leaning broadsheets (*Telegraph*, *Times*) and right-wing tabloids (*Express*, *Daily Mail*, *The Sun*), which stressed how citizens can modify their individual behaviours and habits for a better diet and health^(56,57)^. The political orientation was derived directly from the classifications provided in the identified studies that analysed news content and media coverage^(31,56)^. In addition, other studies stated that individual responsibility for poor diets was stressed more in sensationalist tabloids (tend to be associated with a working-class readership) (*Daily Mail Express*, *Sunday Mirror*, *The Sun*). Middle-class-oriented newspapers highlighted more societal factors (*Guardian*, *Observer*, *Independent* and *Daily Telegraph*)^(31,57)^.

Synthesis of these data also identified that the patterns of responsibility and aetiology have changed over time^(30,31,56)^. From 1996 to 2010, the focal point shifted from individual to societal responsibility^(31)^. However, that trend has not been sustained. According to more recent research, discussion of food industry responsibility has diminished in the news over time, and obesity has been debated less as a political issue^(30)^. Also, the identified studies did not explore news related directly to disadvantaged groups.

### Framing of responsibility in UK government policy

Five articles critically examined the language, framing and narratives used in official UK government policy documents concerning poor diets and associated health consequences among the population. The key finding was that all policy initiatives have predominantly focused on persuading individuals to modify their lifestyle choices and behaviours to reduce their personal health risks, especially through diet changes^(49,58,59,78,79)^, reflecting individual responsibility framing.

Early policy documents (late 20th century/early 21st century) like *Saving Lives: Our Healthier Nation (1999)* or *a report by the Controller and Auditor General (head of the National Audit Office) (2001)* framed obesity as a societal issue targeting the individual consumer as the agent of change^(59)^, emphasising individual responsibility. Later initiatives, such as *Tackling Health Inequalities: A Programme for Action (2003)*, *Choosing Health (2004)* and *Healthy Start (2004)*, positioned the government’s role as facilitating greater consumer understanding of healthy food choices^(49)^. This notion of individual responsibility extended beyond those with health issues to family members like parents. Four of the five articles^(49,58,78,79)^ showed framing included parental responsibility for moderating children’s food advertising exposure^(58)^, acting as role models for healthy behaviours^(49,79)^ or providing nutritional information for parents to make healthier food purchases^(78)^.

Over time, some attempts to acknowledge the importance of external factors of poor diets and diet-related conditions were incorporated into UK government policies, indicating limited recognition of societal responsibility. However, these policies continued to focus on individuals as responsible for poor diets and diet-related conditions without adequately addressing wider environmental or contextual determinants. The following are some key policy developments, though it is not an exhaustive list of all policies during this period. For example, while the *House of Commons Health Committee report on obesity (2004)* recognised the influence of food industry marketing on children, it still targeted individual consumers and parental responsibility as key to making healthier food choices^(59)^. The *Choosing Health: Making Healthy Choices Easier (2004)* policy recognised the industry’s role in educating consumers on a healthy diet, and it committed to future partnerships with food companies to promote this educational effort. Finally, *Tackling Obesities: Future Choices (2007)* reframed obesity as a complex issue requiring system approaches, yet still positioned the individual at the heart of the aetiology and mainly responsible for obesity^(59)^.

Subsequent programmes like *Change4Life (2009)* ^(59,79)^, which were oriented towards encouraging children and their parents to live healthier lives^(79)^, acknowledged the relational nature of obesity and provided educational materials to ‘facilitate’ better lifestyle choices but maintained that rational citizens should take the core action to change^(59)^, reinforcing individual responsibility framing. A broader policy response was initiated in *Tackling Obesity: Empowering Adults and Children to Live Healthier Lives (2020)*, which included environmental measures like calorie labelling in restaurants and a renewed commitment to advertising restrictions. However, much of the framing still emphasised ‘modifiable’ obesity resulting from poor personal choices (which require having information and knowledge)^(58,78)^.

Some of these documents provided limited information that applied to the needs of underserved groups, only referring to health inequalities and socio-economically disadvantaged groups in very few documents^(49,59,78)^. For example, *Saving Lives: Our Healthier Nation (1999)* attributed lower socio-economic groups’ unhealthy eating to individuals’ attitudes and deficiencies in knowledge. Recently, Griffin *et al.* ^(78)^ criticised the *Childhood obesity: a plan for action* (2016, 2018 and 2020) initiative for promoting healthier food choices through nutritional labelling without considering food insecurity and poverty issues like food affordability, food bank usage or energy costs of preparing food^(78)^.

## Discussion

This scoping review systematically synthesised how responsibility for poor diets and obesity is framed across three different sectors in the UK, paying particular attention to how groups experiencing disadvantage are represented. To our knowledge, this study brings new significant insights, extending previous single-domain framing of responsibility reviews^(28,49)^ by providing the first comprehensive picture of responsibility narratives across three main arenas to which people are exposed in their everyday life and that influence people’s perceptions of responsibility for poor diets: public discourse, media coverage and policy domains.

Our findings showed that the responsibility for poor diet and obesity is positioned on individuals, with the exception of the left-wing media, which positioned commercial and environmental factors as key drivers of individual behaviour, which should be addressed through government legislation. The studies selected suggested that more recent government policies have included strategies to address some obesogenic drivers, but these are positioned alongside the ongoing narrative that individuals must take stronger action to improve their dietary choices. Specific reference to groups experiencing disadvantage was rarely made but suggested that individuals experiencing disadvantage require more nutrition education and skills to enact healthier food practices.

Studies examining public perceptions of drivers of food choice showed a multifaceted understanding of the issue, acknowledging both personal and environmental factors. Individual responsibility, however, was emphasised more consistently as the main cause of poor diets, with factors such as a lack of willpower, nutrition knowledge, cooking skills or self-control commonly identified. This finding aligns with previous review results on public perceptions of responsibility for obesity^(81,82)^. Environmental influences, including limited income, food costs, time constraints due to competing demands (e.g. childcare and work schedules), unhealthy food marketing strategies and widespread unhealthy food availability and accessibility, were also acknowledged in the synthesis and are consistent with existing scientific evidence^(81,83–88)^. Studies focusing on groups that experience socio-economic disadvantage particularly highlighted personal factors as the most important driver, while family influences, income and food prices were identified as the most important environmental determinants.

Studies analysing media (press) revealed similar patterns of individual responsibility dominating the narrative. Right-leaning, middle-market and sensationalist newspapers particularly highlighted the need for individual lifestyle changes to address poor diets and diet-related health conditions. In contrast, left-wing newspapers placed more emphasis on the role of the food industry and the government. This pattern differs from that in the European media, with German coverage on sugar taxation showing considerably less focus on individual responsibility than in the UK^(89)^, suggesting that responsibility framing may be culturally situated within political-economic systems. Also, the differences between various types of press outlets suggest that readers are likely to receive different messages about who is responsible for poor diets based on their choice of newspaper. This finding has significant implications, especially for families experiencing socio-economic disadvantage who may have greater exposure to tabloid right-leaning newspapers. These newspapers tend to present obesity and poor diet primarily as individual issues, rarely discussing structural or environmental causes. As a result, readers of these publications may be less likely to support or demand systemic solutions to diet-related health problems^(31)^.

Studies analysing UK government policies consistently frame citizens as holding responsibility for making healthier food choices because such choices can be made rationally and logically. Despite some attempts to recognise environmental drivers over recent years, policies typically focus on providing information and education rather than addressing external factors. This ‘call to action’ approach burdens individuals with the responsibility to modify their dietary practices^(90)^ and may reflect industry influence in policy decision-making^(12–16,91)^. The narratives in government and media documents likely reflect decades of conservative dominance and neoliberal economic policies that emphasise personal choice while minimising state intervention in social issues^(58)^. The policy studies also showed little emphasis on health inequalities, though when mentioned, the narrative of individual responsibility was identified. The emphasis on individual responsibility and efforts to provide more lifestyle information to groups experiencing socio-economic disadvantage is unrealistic, unfair and insufficient to prompt meaningful behaviour change with concurrent efforts to address the environmental determinants of dietary inequalities. Citizens experiencing socio-economic disadvantage lack sufficient financial means and face life circumstances, such as irregular work and shift patterns, which limit their capacity to enact the lifestyle changes they are being implored to make^(56)^.

Our analysis revealed temporal patterns in responsibility framing across mass media (press) and government policies. While not an initial research focus, these chronological variations emerged as noteworthy findings. Mass media (press) framing shifted from individual responsibility to greater emphasis on societal responsibility from 1996 to 2010. Similarly, the framing within government policy evolved from targeting individual behaviour to approaches that, while still emphasising personal responsibility, increasingly acknowledged environmental influences on dietary choices. Studies exploring public perception spanning 2002–2023 show persistent complexity in how responsibility for food choices is attributed, with no clear pattern emerging. Notably, our analysis revealed a striking scarcity of content addressing socio-economically disadvantaged populations across all three domains. Despite our focus on identifying how these groups are represented within responsibility frames, relevant content was limited in both quantity and depth. This absence constitutes an important finding, suggesting that disadvantaged populations remain largely invisible in mainstream diet responsibility discussions, despite bearing a disproportionate burden of diet-related diseases.

### Implications for practice and future research

These findings have significant implications for clarifying the disconnect between scientific evidence and public policy support. Our findings provide crucial evidence of the dominance of individual responsibility narratives across all three domains, which is particularly concerning given that individual responsibility frames effectively reduce public support for health policies^(28)^. This cross-domain consistency may create strong barriers to policy support, as citizens receive reinforcing messages about individual blame from multiple influential sources simultaneously. Despite robust evidence demonstrating that environmental and commercial factors are primary drivers of poor diets^(12,13)^, shifting public opinion towards supporting structural interventions will require coordinated efforts across multiple spheres of influence rather than targeting any single domain.

Participatory approaches with citizens, particularly those experiencing disadvantaged circumstances^(81,82)^, could provide valuable insights into the root causes of poor diets and develop interventions that align with their lived experiences^(92)^. Individuals with lived experience can provide relevant insights into the systematic barriers and social injustices perpetuated by governments^(93)^, mass media^(94)^ and the food industry itself^(95)^, potentially encouraging these powerful structures to consider meaningful change^(96)^. Examples include the youth-led movement Bite Back, which empowers young people to confront the junk food industry through media engagement and parliamentary advocacy; this organisation aims to drive policy changes towards a healthier food environment for young people in England^(97)^. Another example is The Food Conversation, the UK’s largest-ever citizen deliberation on food systems, where people engage with government, business and civil society leaders to suggest interventions addressing system leadership, collaboration, power imbalances, farming fairness and local area potential^(98)^.

The disconnect between scientific evidence and public perception warrants further investigation. Future research should examine the interrelationships between media framing, public opinion and policy development to explore whether these domains operate as mutually reinforcing systems. Priority areas include investigating mechanisms through which food industry actors shape responsibility narratives across domains, using methods such as interviews with citizens, media professionals and commercial sector representatives to better understand these dynamics. This review is a precursor to a future primary qualitative study on public perceptions. Systems mapping with local and national authorities could identify intervention points addressing commercial determinants influencing food practices. Also, implementation studies should examine whether strategic communication approaches can effectively shift public understanding towards greater recognition of societal factors and whether they can build support for structural policies that address the root causes of poor diets^(99)^.

### Study strengths and limitations

This scoping review makes several novel contributions to understanding responsibility framing for poor diets. First, it provides the first systematic synthesis of responsibility framing across multiple domains (public perceptions, mass media and policy) in the UK context. This complete picture demonstrates cross-domain consistency in responsibility framing patterns (revealing consistent individual responsibility emphasis despite evidence of societal drivers) that may help explain barriers to evidence-based policy implementation. Second, it demonstrates a methodological approach for systematically analysing implied responsibility attributions from studies examining multiple factors influencing food choices, thereby broadening the scope of evidence that can inform responsibility framing research. Third, it identifies significant gaps in the representation of disadvantaged groups in this research area, despite their disproportionate burden of diet-related disease.

This review has some limitations. First, it included only peer-reviewed literature, omitting perspectives of non-government or civil society organisations that actively advocate for stronger government policy to curb unhealthy commercial practices. Second, all included articles were UK-based. Including research from countries with similar food environments, such as the USA, could have provided further insights. Third, excluding other stakeholder groups (e.g. healthcare professionals, policymakers) may have missed relevant information. Fourth, our search may have missed studies using alternative terminology for responsibility framing, such as ‘blame’, ‘attribution’, ‘accountability’, ‘culpability’ or ‘agency’. Fifth, our analysis of mass media was limited to newspapers, potentially missing important framing perspectives from non-print media, which are increasingly central to public discourse but appear underrepresented in peer-reviewed literature examining responsibility framing for poor diets. Sixth, a significant limitation of this study relates to our interpretive approach to responsibility attribution. While some included studies directly examined responsibility for poor diets, others investigated factors influencing food choices without explicitly addressing responsibility. For these studies, we applied an interpretive framework to infer responsibility attributions from how factors were framed (e.g. interpreting high food costs as indicating societal responsibility). However, this approach has three key limitations: participants or authors may describe barriers without assigning blame; when external factors are identified, the target of responsibility attribution may vary (e.g. food industry, government policies or economic systems more broadly) in ways that our analysis did not capture; and therefore, our interpretive judgments may not accurately reflect the actual responsibility attributions. Despite these limitations, this review provides a valuable synthesis of evidence on responsibility framing for poor diets in the UK. Future research should incorporate a wider range of sources (e.g. the content of web pages of professional bodies and charities related to obesity and policy databases), expand the geographical scope and explore the perspectives of other stakeholders.

### Conclusions

This scoping review reveals that across the domains of the public, mass media (press) and government policies, poor diet and obesity are almost consistently framed as being an individual responsibility in the UK. While the social and environmental determinants of food choices are acknowledged to some extent, narratives persistently centre on individual responsibility, obscuring the powerful influence of food manufacturers and retailers and the role of government in providing safe, healthy environments for all. There is an urgent need to challenge and reframe this narrative on individual responsibility. The public health nutrition community can and should collectively work towards forcing a radical shift in public, media and policy framing to incite strong regulatory action by governments. Effectively addressing the root causes of diet-related health inequalities will require policymakers to abandon their neoliberal ideology and implement mandatory regulatory frameworks that set standards for commercial practices. Such action would prioritise the health and well-being of all members of society, particularly those most impacted by the burden of poor diets and diet-related diseases.

## Supporting information

Serrano-Fuentes et al. supplementary material 1 Serrano-Fuentes et al. supplementary material

Serrano-Fuentes et al. supplementary material 2 Serrano-Fuentes et al. supplementary material

Serrano-Fuentes et al. supplementary material 3 Serrano-Fuentes et al. supplementary material

## Supplementary material

The supplementary material for this article can be found at https://doi.org/10.1017/S1368980025101079
