# Supplementary material for: Mapping how responsibility for poor diets is framed in the United Kingdom: a scoping review
Source: Public Health Nutr. 2025 Sep 22;28(1):e167. doi: 10.1017/S1368980025101079 (PMC12722098; doi:10.1017/S1368980025101079)
Supplement: Serrano-Fuentes et al. supplementary material 1 — Serrano-Fuentes et al. supplementary material [file S1368980025101079sup001.docx]

**Supplementary material 1**

Table. Participants, Concept and Context (PCC) framework

| **Attributes** | **Description** |
| --- | --- |
| **Participants** | There is a specific focus on citizens pertaining to disadvantaged groups of all ages. For this research, disadvantaged groups are people who experience health inequalities, that is, differences in health across the population that are unfair, avoidable and systematic. These unjust differences are caused by the conditions in which people are born, grow up, live and work ^(1)^, particularly, citizens with characteristics that could be more influenced by the food environment, such as living in deprived areas and having low income and low levels of education, are considered. However, non-disadvantaged groups are also considered if relevant to address the research aim.  The inclusion of all age groups responds to the need to understand the influence of the food environment throughout the life span both to prevent and intervene in the mechanisms of influence of the non-static environment ^(2)^. Only then can the close links between early disadvantage and poor outcomes during the life course be broken ^(3)^.  Public voices (citizens) were included as lay perspectives except for healthcare professionals, policymakers, and food industry staff since these professionals could be more familiar with the influence of commercial actors and the government on diets. These stakeholders operate with specialised knowledge, professional frameworks, and institutional interests that shape how they discuss responsibility for poor diets. Their perspectives would represent an entirely different category of discourse than this study aims to capture - the mainstream public discourse that ordinary citizens encounter and participate in. This creates a clearer picture of how responsibility is framed in the spaces where most people form their opinions. |
| **Concepts** | Poor/unhealthy diets: diets with many carbohydrates, total fats, saturated fats, salt and free sugars, as well as not enough fibre, protein and potassium ^(4)^.  Diet-related conditions: include overweight/obesity, cardiovascular diseases (such as arterial hypertension, stroke, and myocardial infarction), diabetes mellitus type 2, and certain cancers ^(5)^. Maternal obesity is excluded from this study since there are other specific contributing factors that could differ from those of the general population with excess weight.  Social determinants of health: non-medical factors that impact health outcomes. They are the conditions in which people are born, grow, live, work, and age, and the wider set of systems and forces modifying the conditions of daily life; for example: education, income and social protection, working life conditions, food insecurity or housing, basic amenities and the environment ^(6)^.  Obesogenic food environment: settings in which a variety of food is accessible and available to individuals out-of-home and tends to promote obesity ^(7)^. |
| **Context** | Individuals were considered to be embedded in a multilevel system where the interactive characteristics of individuals and environments underlie health results ^(8)^. In the analysis, multiple levels ^(2,9,10)^ helped systematically categorise the diverse range of influences on food choices identified across studies (see Supplementary material 3).  The country considered was the UK. It is essential to focus on the characteristics of the country studied since the literature suggests the existence of different profiles of diet-related conditions and risk factors operating in different countries ^(11)^. Also, the characteristics of the food environment in the UK might differ from those of other developed countries. |

**References**

1. National Institute for Health and Care Excellence [NICE] (2023) NICE and health inequalities | What we do | About. https://www.nice.org.uk/about/what-we-do/nice-and-health-inequalities (accessed November 2023).

2. Serrano Fuentes N (2023) Exploring the impact of multilevel environments influencing the adoption of health practices in adults with obesity in the United Kingdom. phd, University of Southampton.

3. Marmot M (2010) *Fair Society, Healthy Lives: the Marmot Review: strategic review of health inequalities in England post-2010*. .

4. Lang T (2020) *Feeding Britain. Our Food Problems and How to Fix Them.* Dublin: Penguin Random House UK.

5. World Health Organization [WHO] (2024) Fact sheets - Malnutrition. https://www.who.int/news-room/fact-sheets/detail/malnutrition (accessed May 2024).

6. World Health Organization [WHO] (2024) Social determinants of health. https://www.who.int/health-topics/social-determinants-of-health (accessed February 2024).

7. Swinburn B, Egger G & Raza F (1999) Dissecting obesogenic environments: the development and application of a framework for identifying and prioritizing environmental interventions for obesity. *Prev Med* **29**, 563–570.

8. Sallis JF, Owen N & Fisher EB (2008) Ecological models of health behavior. In *Health behavior and health education: Theory, research, and practice.*, 4th ed., pp. 465–485 [Glanz K, Rimer BK, Viswanath K, editors]. San Francisco, CA, US: Jossey-Bass.

9. Bronfenbrenner U (1979) *The Ecology of Human Development: Experiments by Nature and Design*. United States: Harvard University Press.

10. Dahlgren G & Whitehead M (1991) *Policies and strategies to promote social equity in health*. Stockholm, Sweden: Institute for Futures Studies.

11. Blundell JE, Baker JL, Boyland E, et al. (2017) Variations in the Prevalence of Obesity Among European Countries, and a Consideration of Possible Causes. *Obesity Facts* **10**, 25–37.
