# Supplementary material for: Mapping how responsibility for poor diets is framed in the United Kingdom: a scoping review
Source: Public Health Nutr. 2025 Sep 22;28(1):e167. doi: 10.1017/S1368980025101079 (PMC12722098; doi:10.1017/S1368980025101079)
Supplement: Serrano-Fuentes et al. supplementary material 2 — Serrano-Fuentes et al. supplementary material [file S1368980025101079sup002.docx]

**Supplementary material 2. Search strategy**

**MEDLINE (Ovid)**

| Set | Search Statement | Annotations | Insert | Edit | Delete |
| --- | --- | --- | --- | --- | --- |
| 1. | ((food or nutrition* or community or consum* or eating or store or obes* or built) adj environment).ab,kf,kw,ti. |  |  |  |  |
| 2. | food industry.ab,kf,kw,ti. |  |  |  |  |
| 3. | food supply.ab,kf,kw,ti. |  |  |  |  |
| 4. | (food adj1 (accept* or access* or acqui* or ad or ads or advertis* or aesthetic* or afford* or attitude* or availab* or brand* or choice* or composition or consumption or convenience or cost* or cultur* or deliver* or desir* or distribut* or knowledge* or label* or marketing or outlet* or packag* or perception* or place* or practice* or preference* or prepar* or price* or pricing* or process* or promot* or provision* or purchas* or quality or retail or sale* or selection or service* or shop* or sponsorship* or stall* or store* or tast* or vendor*)).ab,kf,kw,ti. |  |  |  |  |
| 5. | diet.ab,kf,kw,ti. |  |  |  |  |
| 6. | (feeding adj (behavior or behaviour)).ab,kf,kw,ti. |  |  |  |  |
| 7. | food preferences.ab,kf,kw,ti. |  |  |  |  |
| 8. | (cafe* or canteen* or restaurant* or supermarket* or takeaway* or take-away or vending machine* or kiosk*).ab,kf,kw,ti. |  |  |  |  |
| 9. | (grocery adj (shop* or store*)).ab,kf,kw,ti. |  |  |  |  |
| 10. | (food adj (desert* or swamp*)).ab,kf,kw,ti. |  |  |  |  |
| 11. | (food porn or foodporn or gastroporn).ab,kf,kw,ti. |  |  |  |  |
| 12. | "foodscape* ".ab,kf,kw,ti. |  |  |  |  |
| 13. | "commercial determinants".ab,kf,kw,ti. |  |  |  |  |
| 14. | Fast foods.ab,kf,kw,ti. |  |  |  |  |
| 15. | ((Ultra processed or ultra-processed or processed or "high in fat, salt and sugar" or junk) adj food).ab,kf,kw,ti. |  |  |  |  |
| 16. | ((nutrition or obesity) adj policy).ab,kf,kw,ti. |  |  |  |  |
| 17. | ((obesity or food) adj policy making).ab,kf,kw,ti. |  |  |  |  |
| 18. | ((obesity or food) adj strategic plan*).ab,kf,kw,ti. |  |  |  |  |
| 19. | ((obes* or food) adj news).ab,kf,kw,ti. |  |  |  |  |
| 20. | (food adj3 (activism or collaborat* or partnership or movement*)).ab,kf,kw,ti. |  |  |  |  |
| 21. | 1 or 2 or 3 or 4 or 5 or 6 or 7 or 8 or 9 or 10 or 11 or 12 or 13 or 14 or 15 or 16 or 17 or 18 or 19 or 20 |  |  |  |  |
| 22. | internet.ab,kf,kw,ti. |  |  |  |  |
| 23. | webcasts.ab,kf,kw,ti. |  |  |  |  |
| 24. | web browser.ab,kf,kw,ti. |  |  |  |  |
| 25. | video games.ab,kf,kw,ti. |  |  |  |  |
| 26. | virtual reality.ab,kf,kw,ti. |  |  |  |  |
| 27. | social media.ab,kf,kw,ti. |  |  |  |  |
| 28. | smartphone.ab,kf,kw,ti. |  |  |  |  |
| 29. | online social networking.ab,kf,kw,ti. |  |  |  |  |
| 30. | mobile applications.ab,kf,kw,ti. |  |  |  |  |
| 31. | electronic mail.ab,kf,kw,ti. |  |  |  |  |
| 32. | "cell phone use".ab,kf,kw,ti. |  |  |  |  |
| 33. | cell phone.ab,kf,kw,ti. |  |  |  |  |
| 34. | blogging.ab,kf,kw,ti. |  |  |  |  |
| 35. | (blog* or microblog*).ab,kf,kw,ti. |  |  |  |  |
| 36. | (cyber* or virtual or digital*).ab,kf,kw,ti. |  |  |  |  |
| 37. | ((digital or new) adj media).ab,kf,kw,ti. |  |  |  |  |
| 38. | "information technolog* ".ab,kf,kw,ti. |  |  |  |  |
| 39. | (smartphone* or mobile* or touchscreen* or wearable*).ab,kf,kw,ti. |  |  |  |  |
| 40. | (social adj (media or network*)).ab,kf,kw,ti. |  |  |  |  |
| 41. | (web-based or online or on-line).ab,kf,kw,ti. |  |  |  |  |
| 42. | (website* or web site* or webpage* or web page*).ab,kf,kw,ti. |  |  |  |  |
| 43. | (Facebook or Instagram* or Twitter or tweet* or Snapchat or YouTube or Reddit or WhatsApp or TikTok or Tumblr or Pinterest or LinkedIn).ab,kf,kw,ti. |  |  |  |  |
| 44. | 22 or 23 or 24 or 25 or 26 or 27 or 28 or 29 or 30 or 31 or 32 or 33 or 34 or 35 or 36 or 37 or 38 or 39 or 40 or 41 or 42 or 43 |  |  |  |  |
| 45. | 21 and 44 |  |  |  |  |
| 46. | 21 or 45 |  |  |  |  |
| 47. | (responsib* adj1 (obesity or overweight or "excess weight" or diet* or nutrit*)).ab,kf,kw,ti. |  |  |  |  |
| 48. | (caus* adj2 (obesity or overweight or "excess weight" or diet* or nutrit*)).ab,kf,kw,ti. |  |  |  |  |
| 49. | (discourse* adj1 (obesity or overweight or "excess weight" or diet* or nutrit*)).ab,kf,kw,ti. |  |  |  |  |
| 50. | (framing adj (obesity or overweight or "excess weight" or diet* or nutrit*)).ab,kf,kw,ti. |  |  |  |  |
| 51. | (obesity adj1 ("life experience" or experience or belief* or voice* or "living with")).ab,kf,kw,ti. |  |  |  |  |
| 52. | 47 or 48 or 49 or 50 or 51 |  |  |  |  |
| 53. | 21 or 45 or 52 |  |  |  |  |
| 54. | (gb or great Britain or britain or british or uk or united kingdom or england or english or northern ireland or northern irish or scotland or scottish or wales or welsh).ab,kf,kw,ti,tw. |  |  |  |  |
| 55. | 53 and 54 |  |  |  |  |
| 56. | limit 55 to (yr="2000 -Current" and english) |  |  |  |  |

| **CINAHL (EBSCOhost)** | Sunday, January 21, 2024 3:12:24 PM |
| --- | --- |

| **#** | **Query** | **Limiters/Expanders** | **Last Run Via** | **Results** |
| --- | --- | --- | --- | --- |
| S35 | S31 AND S32 | Limiters - Publication Date: 20000101-20231231 Expanders - Apply equivalent subjects Narrow by Language: - english Search modes - Find all my search terms | Interface - EBSCOhost Research Databases Search Screen - Advanced Search Database - CINAHL Plus with Full Text | Display |
| S34 | S31 AND S32 | Limiters - Publication Date: 20000101-20231231 Expanders - Apply equivalent subjects Search modes - Find all my search terms | Interface - EBSCOhost Research Databases Search Screen - Advanced Search Database - CINAHL Plus with Full Text | Display |
| S33 | S31 AND S32 | Expanders - Apply equivalent subjects Search modes - Find all my search terms | Interface - EBSCOhost Research Databases Search Screen - Advanced Search Database - CINAHL Plus with Full Text | Display |
| S32 | TI ( united kingdom or uk or britain or scotland or england or wales or northern ireland ) OR AB ( united kingdom or uk or britain or scotland or england or wales or northern ireland ) | Expanders - Apply equivalent subjects Search modes - Find all my search terms | Interface - EBSCOhost Research Databases Search Screen - Advanced Search Database - CINAHL Plus with Full Text | Display |
| S31 | S20 OR S30 | Expanders - Apply equivalent subjects Search modes - Find all my search terms | Interface - EBSCOhost Research Databases Search Screen - Advanced Search Database - CINAHL Plus with Full Text | Display |
| S30 | S26 OR S27 OR S28 OR S29 | Expanders - Apply equivalent subjects Search modes - Find all my search terms | Interface - EBSCOhost Research Databases Search Screen - Advanced Search Database - CINAHL Plus with Full Text | Display |
| S29 | S21 AND S25 | Expanders - Apply equivalent subjects Search modes - Find all my search terms | Interface - EBSCOhost Research Databases Search Screen - Advanced Search Database - CINAHL Plus with Full Text | Display |
| S28 | S21 AND S24 | Expanders - Apply equivalent subjects Search modes - Find all my search terms | Interface - EBSCOhost Research Databases Search Screen - Advanced Search Database - CINAHL Plus with Full Text | Display |
| S27 | S21 AND S23 | Expanders - Apply equivalent subjects Search modes - Find all my search terms | Interface - EBSCOhost Research Databases Search Screen - Advanced Search Database - CINAHL Plus with Full Text | Display |
| S26 | S21 AND S22 | Expanders - Apply equivalent subjects Search modes - Find all my search terms | Interface - EBSCOhost Research Databases Search Screen - Advanced Search Database - CINAHL Plus with Full Text | Display |
| S25 | TI ( causes or factors or determinants or factors or contributing factors or determining factors ) OR AB ( causes or factors or determinants or factors or contributing factors or determining factors ) | Expanders - Apply equivalent subjects Search modes - Find all my search terms | Interface - EBSCOhost Research Databases Search Screen - Advanced Search Database - CINAHL Plus with Full Text | Display |
| S24 | TI discourse OR AB discourse | Expanders - Apply equivalent subjects Search modes - Find all my search terms | Interface - EBSCOhost Research Databases Search Screen - Advanced Search Database - CINAHL Plus with Full Text | Display |
| S23 | TI ( responsibility or accountability ) OR AB ( responsibility or accountability ) | Expanders - Apply equivalent subjects Search modes - Find all my search terms | Interface - EBSCOhost Research Databases Search Screen - Advanced Search Database - CINAHL Plus with Full Text | Display |
| S22 | TI ( experiences or perceptions or attitudes or views or feelings or qualitative or perspective ) OR AB ( experiences or perceptions or attitudes or views or feelings or qualitative or perspective ) | Expanders - Apply equivalent subjects Search modes - Find all my search terms | Interface - EBSCOhost Research Databases Search Screen - Advanced Search Database - CINAHL Plus with Full Text | Display |
| S21 | TI ( obesity or overweight or fat or obese or unhealthy weight or high bmi or high body mass index ) OR AB ( obesity or overweight or fat or obese or unhealthy weight or high bmi or high body mass index ) | Expanders - Apply equivalent subjects Search modes - Find all my search terms | Interface - EBSCOhost Research Databases Search Screen - Advanced Search Database - CINAHL Plus with Full Text | Display |
| S20 | S14 OR S19 | Expanders - Apply equivalent subjects Search modes - Find all my search terms | Interface - EBSCOhost Research Databases Search Screen - Advanced Search Database - CINAHL Plus with Full Text | Display |
| S19 | S14 AND S18 | Expanders - Apply equivalent subjects Search modes - Find all my search terms | Interface - EBSCOhost Research Databases Search Screen - Advanced Search Database - CINAHL Plus with Full Text | Display |
| S18 | S15 OR S16 OR S17 | Expanders - Apply equivalent subjects Search modes - Find all my search terms | Interface - EBSCOhost Research Databases Search Screen - Advanced Search Database - CINAHL Plus with Full Text | Display |
| S17 | TI ( facebook or twitter or instagram or snapchat or tumblr or tiktok or youtube ) OR AB ( facebook or twitter or instagram or snapchat or tumblr or tiktok or youtube ) | Expanders - Apply equivalent subjects Search modes - Find all my search terms | Interface - EBSCOhost Research Databases Search Screen - Advanced Search Database - CINAHL Plus with Full Text | Display |
| S16 | TI ( app or mobile app or apps or mobile device applications or mobile apps or smartphone ) OR AB ( app or mobile app or apps or mobile device applications or mobile apps or smartphone ) | Expanders - Apply equivalent subjects Search modes - Find all my search terms | Interface - EBSCOhost Research Databases Search Screen - Advanced Search Database - CINAHL Plus with Full Text | Display |
| S15 | TI ( online or internet or web or social media ) OR AB ( online or internet or web or social media ) | Expanders - Apply equivalent subjects Search modes - Find all my search terms | Interface - EBSCOhost Research Databases Search Screen - Advanced Search Database - CINAHL Plus with Full Text | Display |
| S14 | S1 OR S2 OR S3 OR S4 OR S5 OR S6 OR S7 OR S8 OR S9 OR S10 OR S11 OR S12 OR S13 | Expanders - Apply equivalent subjects Search modes - Find all my search terms | Interface - EBSCOhost Research Databases Search Screen - Advanced Search Database - CINAHL Plus with Full Text | Display |
| S13 | TI food business OR AB food business | Expanders - Apply equivalent subjects Search modes - Find all my search terms | Interface - EBSCOhost Research Databases Search Screen - Advanced Search Database - CINAHL Plus with Full Text | Display |
| S12 | TI food activism OR AB food activism | Expanders - Apply equivalent subjects Search modes - Find all my search terms | Interface - EBSCOhost Research Databases Search Screen - Advanced Search Database - CINAHL Plus with Full Text | Display |
| S11 | AB ( news media or news or news coverage or journalism ) AND AB obesity | Expanders - Apply equivalent subjects Search modes - Find all my search terms | Interface - EBSCOhost Research Databases Search Screen - Advanced Search Database - CINAHL Plus with Full Text | Display |
| S10 | TI food policy OR AB food policy | Expanders - Apply equivalent subjects Search modes - Find all my search terms | Interface - EBSCOhost Research Databases Search Screen - Advanced Search Database - CINAHL Plus with Full Text | Display |
| S9 | TI obesity policy OR AB obesity policy | Expanders - Apply equivalent subjects Search modes - Find all my search terms | Interface - EBSCOhost Research Databases Search Screen - Advanced Search Database - CINAHL Plus with Full Text | Display |
| S8 | TI takeaway food OR AB takeaway food | Expanders - Apply equivalent subjects Search modes - Find all my search terms | Interface - EBSCOhost Research Databases Search Screen - Advanced Search Database - CINAHL Plus with Full Text | Display |
| S7 | TI ( ultra-processed foods or junk food or fast food ) OR AB ( ultra-processed foods or junk food or fast food ) | Expanders - Apply equivalent subjects Search modes - Find all my search terms | Interface - EBSCOhost Research Databases Search Screen - Advanced Search Database - CINAHL Plus with Full Text | Display |
| S6 | TI commercial determinants of health OR AB commercial determinants of health | Expanders - Apply equivalent subjects Search modes - Find all my search terms | Interface - EBSCOhost Research Databases Search Screen - Advanced Search Database - CINAHL Plus with Full Text | Display |
| S5 | TI food marketing OR AB food marketing | Expanders - Apply equivalent subjects Search modes - Find all my search terms | Interface - EBSCOhost Research Databases Search Screen - Advanced Search Database - CINAHL Plus with Full Text | Display |
| S4 | TI food industry OR AB food industry | Expanders - Apply equivalent subjects Search modes - Find all my search terms | Interface - EBSCOhost Research Databases Search Screen - Advanced Search Database - CINAHL Plus with Full Text | Display |
| S3 | TI ( supermarkets or grocery stores or food retail ) OR AB ( supermarkets or grocery stores or food retail ) | Expanders - Apply equivalent subjects Search modes - Find all my search terms | Interface - EBSCOhost Research Databases Search Screen - Advanced Search Database - CINAHL Plus with Full Text | Display |
| S2 | TI obesogenic environment OR AB obesogenic environment | Expanders - Apply equivalent subjects Search modes - Find all my search terms | Interface - EBSCOhost Research Databases Search Screen - Advanced Search Database - CINAHL Plus with Full Text | Display |
| S1 | TI ( food environment or built food environment ) OR AB ( food environment or built food environment ) | Expanders - Apply equivalent subjects Search modes - Find all my search terms | Interface - EBSCOhost Research Databases Search Screen - Advanced Search Database - CINAHL Plus with Full Text | Display |

**PsycINFO (EBSCOhost)**

| S35 | S31 AND S32 | Limiters - Publication Date: 20000101-20231231 Expanders - Apply equivalent subjects Narrow by Language: - english Search modes - Find all my search terms | Interface - EBSCOhost Research Databases Search Screen - Advanced Search Database - APA PsycInfo | Display |
| --- | --- | --- | --- | --- |
| S34 | S31 AND S32 | Limiters - Publication Date: 20000101-20231231 Expanders - Apply equivalent subjects Search modes - Find all my search terms | Interface - EBSCOhost Research Databases Search Screen - Advanced Search Database - CINAHL Plus with Full Text | Display |
| S33 | S31 AND S32 | Expanders - Apply equivalent subjects Search modes - Find all my search terms | Interface - EBSCOhost Research Databases Search Screen - Advanced Search Database - CINAHL Plus with Full Text | Display |
| S32 | TI ( united kingdom or uk or britain or scotland or england or wales or northern ireland ) OR AB ( united kingdom or uk or britain or scotland or england or wales or northern ireland ) | Expanders - Apply equivalent subjects Search modes - Find all my search terms | Interface - EBSCOhost Research Databases Search Screen - Advanced Search Database - CINAHL Plus with Full Text | Display |
| S31 | S20 OR S30 | Expanders - Apply equivalent subjects Search modes - Find all my search terms | Interface - EBSCOhost Research Databases Search Screen - Advanced Search Database - CINAHL Plus with Full Text | Display |
| S30 | S26 OR S27 OR S28 OR S29 | Expanders - Apply equivalent subjects Search modes - Find all my search terms | Interface - EBSCOhost Research Databases Search Screen - Advanced Search Database - CINAHL Plus with Full Text | Display |
| S29 | S21 AND S25 | Expanders - Apply equivalent subjects Search modes - Find all my search terms | Interface - EBSCOhost Research Databases Search Screen - Advanced Search Database - CINAHL Plus with Full Text | Display |
| S28 | S21 AND S24 | Expanders - Apply equivalent subjects Search modes - Find all my search terms | Interface - EBSCOhost Research Databases Search Screen - Advanced Search Database - CINAHL Plus with Full Text | Display |
| S27 | S21 AND S23 | Expanders - Apply equivalent subjects Search modes - Find all my search terms | Interface - EBSCOhost Research Databases Search Screen - Advanced Search Database - CINAHL Plus with Full Text | Display |
| S26 | S21 AND S22 | Expanders - Apply equivalent subjects Search modes - Find all my search terms | Interface - EBSCOhost Research Databases Search Screen - Advanced Search Database - CINAHL Plus with Full Text | Display |
| S25 | TI ( causes or factors or determinants or factors or contributing factors or determining factors ) OR AB ( causes or factors or determinants or factors or contributing factors or determining factors ) | Expanders - Apply equivalent subjects Search modes - Find all my search terms | Interface - EBSCOhost Research Databases Search Screen - Advanced Search Database - CINAHL Plus with Full Text | Display |
| S24 | TI discourse OR AB discourse | Expanders - Apply equivalent subjects Search modes - Find all my search terms | Interface - EBSCOhost Research Databases Search Screen - Advanced Search Database - CINAHL Plus with Full Text | Display |
| S23 | TI ( responsibility or accountability ) OR AB ( responsibility or accountability ) | Expanders - Apply equivalent subjects Search modes - Find all my search terms | Interface - EBSCOhost Research Databases Search Screen - Advanced Search Database - CINAHL Plus with Full Text | Display |
| S22 | TI ( experiences or perceptions or attitudes or views or feelings or qualitative or perspective ) OR AB ( experiences or perceptions or attitudes or views or feelings or qualitative or perspective ) | Expanders - Apply equivalent subjects Search modes - Find all my search terms | Interface - EBSCOhost Research Databases Search Screen - Advanced Search Database - CINAHL Plus with Full Text | Display |
| S21 | TI ( obesity or overweight or fat or obese or unhealthy weight or high bmi or high body mass index ) OR AB ( obesity or overweight or fat or obese or unhealthy weight or high bmi or high body mass index ) | Expanders - Apply equivalent subjects Search modes - Find all my search terms | Interface - EBSCOhost Research Databases Search Screen - Advanced Search Database - CINAHL Plus with Full Text | Display |
| S20 | S14 OR S19 | Expanders - Apply equivalent subjects Search modes - Find all my search terms | Interface - EBSCOhost Research Databases Search Screen - Advanced Search Database - CINAHL Plus with Full Text | Display |
| S19 | S14 AND S18 | Expanders - Apply equivalent subjects Search modes - Find all my search terms | Interface - EBSCOhost Research Databases Search Screen - Advanced Search Database - CINAHL Plus with Full Text | Display |
| S18 | S15 OR S16 OR S17 | Expanders - Apply equivalent subjects Search modes - Find all my search terms | Interface - EBSCOhost Research Databases Search Screen - Advanced Search Database - CINAHL Plus with Full Text | Display |
| S17 | TI ( facebook or twitter or instagram or snapchat or tumblr or tiktok or youtube ) OR AB ( facebook or twitter or instagram or snapchat or tumblr or tiktok or youtube ) | Expanders - Apply equivalent subjects Search modes - Find all my search terms | Interface - EBSCOhost Research Databases Search Screen - Advanced Search Database - CINAHL Plus with Full Text | Display |
| S16 | TI ( app or mobile app or apps or mobile device applications or mobile apps or smartphone ) OR AB ( app or mobile app or apps or mobile device applications or mobile apps or smartphone ) | Expanders - Apply equivalent subjects Search modes - Find all my search terms | Interface - EBSCOhost Research Databases Search Screen - Advanced Search Database - CINAHL Plus with Full Text | Display |
| S15 | TI ( online or internet or web or social media ) OR AB ( online or internet or web or social media ) | Expanders - Apply equivalent subjects Search modes - Find all my search terms | Interface - EBSCOhost Research Databases Search Screen - Advanced Search Database - CINAHL Plus with Full Text | Display |
| S14 | S1 OR S2 OR S3 OR S4 OR S5 OR S6 OR S7 OR S8 OR S9 OR S10 OR S11 OR S12 OR S13 | Expanders - Apply equivalent subjects Search modes - Find all my search terms | Interface - EBSCOhost Research Databases Search Screen - Advanced Search Database - CINAHL Plus with Full Text | Display |
| S13 | TI food business OR AB food business | Expanders - Apply equivalent subjects Search modes - Find all my search terms | Interface - EBSCOhost Research Databases Search Screen - Advanced Search Database - CINAHL Plus with Full Text | Display |
| S12 | TI food activism OR AB food activism | Expanders - Apply equivalent subjects Search modes - Find all my search terms | Interface - EBSCOhost Research Databases Search Screen - Advanced Search Database - CINAHL Plus with Full Text | Display |
| S11 | AB ( news media or news or news coverage or journalism ) AND AB obesity | Expanders - Apply equivalent subjects Search modes - Find all my search terms | Interface - EBSCOhost Research Databases Search Screen - Advanced Search Database - CINAHL Plus with Full Text | Display |
| S10 | TI food policy OR AB food policy | Expanders - Apply equivalent subjects Search modes - Find all my search terms | Interface - EBSCOhost Research Databases Search Screen - Advanced Search Database - CINAHL Plus with Full Text | Display |
| S9 | TI obesity policy OR AB obesity policy | Expanders - Apply equivalent subjects Search modes - Find all my search terms | Interface - EBSCOhost Research Databases Search Screen - Advanced Search Database - CINAHL Plus with Full Text | Display |
| S8 | TI takeaway food OR AB takeaway food | Expanders - Apply equivalent subjects Search modes - Find all my search terms | Interface - EBSCOhost Research Databases Search Screen - Advanced Search Database - CINAHL Plus with Full Text | Display |
| S7 | TI ( ultra-processed foods or junk food or fast food ) OR AB ( ultra-processed foods or junk food or fast food ) | Expanders - Apply equivalent subjects Search modes - Find all my search terms | Interface - EBSCOhost Research Databases Search Screen - Advanced Search Database - CINAHL Plus with Full Text | Display |
| S6 | TI commercial determinants of health OR AB commercial determinants of health | Expanders - Apply equivalent subjects Search modes - Find all my search terms | Interface - EBSCOhost Research Databases Search Screen - Advanced Search Database - CINAHL Plus with Full Text | Display |
| S5 | TI food marketing OR AB food marketing | Expanders - Apply equivalent subjects Search modes - Find all my search terms | Interface - EBSCOhost Research Databases Search Screen - Advanced Search Database - CINAHL Plus with Full Text | Display |
| S4 | TI food industry OR AB food industry | Expanders - Apply equivalent subjects Search modes - Find all my search terms | Interface - EBSCOhost Research Databases Search Screen - Advanced Search Database - CINAHL Plus with Full Text | Display |
| S3 | TI ( supermarkets or grocery stores or food retail ) OR AB ( supermarkets or grocery stores or food retail ) | Expanders - Apply equivalent subjects Search modes - Find all my search terms | Interface - EBSCOhost Research Databases Search Screen - Advanced Search Database - CINAHL Plus with Full Text | Display |
| S2 | TI obesogenic environment OR AB obesogenic environment | Expanders - Apply equivalent subjects Search modes - Find all my search terms | Interface - EBSCOhost Research Databases Search Screen - Advanced Search Database - CINAHL Plus with Full Text | Display |
| S1 | TI ( food environment or built food environment ) OR AB ( food environment or built food environment ) | Expanders - Apply equivalent subjects Search modes - Find all my search terms | Interface - EBSCOhost Research Databases Search Screen - Advanced Search Database - CINAHL Plus with Full Text | Display |

**ECONLIT (EBSCOhost)**

| S35 | S31 AND S32 | Limiters - Publication Date: 20000101-20231231 Expanders - Apply equivalent subjects Narrow by Language: - english Search modes - Find all my search terms | Interface - EBSCOhost Research Databases Search Screen - Advanced Search Database - EconLit | Display |
| --- | --- | --- | --- | --- |
| S34 | S31 AND S32 | Limiters - Publication Date: 20000101-20231231 Expanders - Apply equivalent subjects Search modes - Find all my search terms | Interface - EBSCOhost Research Databases Search Screen - Advanced Search Database - CINAHL Plus with Full Text | Display |
| S33 | S31 AND S32 | Expanders - Apply equivalent subjects Search modes - Find all my search terms | Interface - EBSCOhost Research Databases Search Screen - Advanced Search Database - CINAHL Plus with Full Text | Display |
| S32 | TI ( united kingdom or uk or britain or scotland or england or wales or northern ireland ) OR AB ( united kingdom or uk or britain or scotland or england or wales or northern ireland ) | Expanders - Apply equivalent subjects Search modes - Find all my search terms | Interface - EBSCOhost Research Databases Search Screen - Advanced Search Database - CINAHL Plus with Full Text | Display |
| S31 | S20 OR S30 | Expanders - Apply equivalent subjects Search modes - Find all my search terms | Interface - EBSCOhost Research Databases Search Screen - Advanced Search Database - CINAHL Plus with Full Text | Display |
| S30 | S26 OR S27 OR S28 OR S29 | Expanders - Apply equivalent subjects Search modes - Find all my search terms | Interface - EBSCOhost Research Databases Search Screen - Advanced Search Database - CINAHL Plus with Full Text | Display |
| S29 | S21 AND S25 | Expanders - Apply equivalent subjects Search modes - Find all my search terms | Interface - EBSCOhost Research Databases Search Screen - Advanced Search Database - CINAHL Plus with Full Text | Display |
| S28 | S21 AND S24 | Expanders - Apply equivalent subjects Search modes - Find all my search terms | Interface - EBSCOhost Research Databases Search Screen - Advanced Search Database - CINAHL Plus with Full Text | Display |
| S27 | S21 AND S23 | Expanders - Apply equivalent subjects Search modes - Find all my search terms | Interface - EBSCOhost Research Databases Search Screen - Advanced Search Database - CINAHL Plus with Full Text | Display |
| S26 | S21 AND S22 | Expanders - Apply equivalent subjects Search modes - Find all my search terms | Interface - EBSCOhost Research Databases Search Screen - Advanced Search Database - CINAHL Plus with Full Text | Display |
| S25 | TI ( causes or factors or determinants or factors or contributing factors or determining factors ) OR AB ( causes or factors or determinants or factors or contributing factors or determining factors ) | Expanders - Apply equivalent subjects Search modes - Find all my search terms | Interface - EBSCOhost Research Databases Search Screen - Advanced Search Database - CINAHL Plus with Full Text | Display |
| S24 | TI discourse OR AB discourse | Expanders - Apply equivalent subjects Search modes - Find all my search terms | Interface - EBSCOhost Research Databases Search Screen - Advanced Search Database - CINAHL Plus with Full Text | Display |
| S23 | TI ( responsibility or accountability ) OR AB ( responsibility or accountability ) | Expanders - Apply equivalent subjects Search modes - Find all my search terms | Interface - EBSCOhost Research Databases Search Screen - Advanced Search Database - CINAHL Plus with Full Text | Display |
| S22 | TI ( experiences or perceptions or attitudes or views or feelings or qualitative or perspective ) OR AB ( experiences or perceptions or attitudes or views or feelings or qualitative or perspective ) | Expanders - Apply equivalent subjects Search modes - Find all my search terms | Interface - EBSCOhost Research Databases Search Screen - Advanced Search Database - CINAHL Plus with Full Text | Display |
| S21 | TI ( obesity or overweight or fat or obese or unhealthy weight or high bmi or high body mass index ) OR AB ( obesity or overweight or fat or obese or unhealthy weight or high bmi or high body mass index ) | Expanders - Apply equivalent subjects Search modes - Find all my search terms | Interface - EBSCOhost Research Databases Search Screen - Advanced Search Database - CINAHL Plus with Full Text | Display |
| S20 | S14 OR S19 | Expanders - Apply equivalent subjects Search modes - Find all my search terms | Interface - EBSCOhost Research Databases Search Screen - Advanced Search Database - CINAHL Plus with Full Text | Display |
| S19 | S14 AND S18 | Expanders - Apply equivalent subjects Search modes - Find all my search terms | Interface - EBSCOhost Research Databases Search Screen - Advanced Search Database - CINAHL Plus with Full Text | Display |
| S18 | S15 OR S16 OR S17 | Expanders - Apply equivalent subjects Search modes - Find all my search terms | Interface - EBSCOhost Research Databases Search Screen - Advanced Search Database - CINAHL Plus with Full Text | Display |
| S17 | TI ( facebook or twitter or instagram or snapchat or tumblr or tiktok or youtube ) OR AB ( facebook or twitter or instagram or snapchat or tumblr or tiktok or youtube ) | Expanders - Apply equivalent subjects Search modes - Find all my search terms | Interface - EBSCOhost Research Databases Search Screen - Advanced Search Database - CINAHL Plus with Full Text | Display |
| S16 | TI ( app or mobile app or apps or mobile device applications or mobile apps or smartphone ) OR AB ( app or mobile app or apps or mobile device applications or mobile apps or smartphone ) | Expanders - Apply equivalent subjects Search modes - Find all my search terms | Interface - EBSCOhost Research Databases Search Screen - Advanced Search Database - CINAHL Plus with Full Text | Display |
| S15 | TI ( online or internet or web or social media ) OR AB ( online or internet or web or social media ) | Expanders - Apply equivalent subjects Search modes - Find all my search terms | Interface - EBSCOhost Research Databases Search Screen - Advanced Search Database - CINAHL Plus with Full Text | Display |
| S14 | S1 OR S2 OR S3 OR S4 OR S5 OR S6 OR S7 OR S8 OR S9 OR S10 OR S11 OR S12 OR S13 | Expanders - Apply equivalent subjects Search modes - Find all my search terms | Interface - EBSCOhost Research Databases Search Screen - Advanced Search Database - CINAHL Plus with Full Text | Display |
| S13 | TI food business OR AB food business | Expanders - Apply equivalent subjects Search modes - Find all my search terms | Interface - EBSCOhost Research Databases Search Screen - Advanced Search Database - CINAHL Plus with Full Text | Display |
| S12 | TI food activism OR AB food activism | Expanders - Apply equivalent subjects Search modes - Find all my search terms | Interface - EBSCOhost Research Databases Search Screen - Advanced Search Database - CINAHL Plus with Full Text | Display |
| S11 | AB ( news media or news or news coverage or journalism ) AND AB obesity | Expanders - Apply equivalent subjects Search modes - Find all my search terms | Interface - EBSCOhost Research Databases Search Screen - Advanced Search Database - CINAHL Plus with Full Text | Display |
| S10 | TI food policy OR AB food policy | Expanders - Apply equivalent subjects Search modes - Find all my search terms | Interface - EBSCOhost Research Databases Search Screen - Advanced Search Database - CINAHL Plus with Full Text | Display |
| S9 | TI obesity policy OR AB obesity policy | Expanders - Apply equivalent subjects Search modes - Find all my search terms | Interface - EBSCOhost Research Databases Search Screen - Advanced Search Database - CINAHL Plus with Full Text | Display |
| S8 | TI takeaway food OR AB takeaway food | Expanders - Apply equivalent subjects Search modes - Find all my search terms | Interface - EBSCOhost Research Databases Search Screen - Advanced Search Database - CINAHL Plus with Full Text | Display |
| S7 | TI ( ultra-processed foods or junk food or fast food ) OR AB ( ultra-processed foods or junk food or fast food ) | Expanders - Apply equivalent subjects Search modes - Find all my search terms | Interface - EBSCOhost Research Databases Search Screen - Advanced Search Database - CINAHL Plus with Full Text | Display |
| S6 | TI commercial determinants of health OR AB commercial determinants of health | Expanders - Apply equivalent subjects Search modes - Find all my search terms | Interface - EBSCOhost Research Databases Search Screen - Advanced Search Database - CINAHL Plus with Full Text | Display |
| S5 | TI food marketing OR AB food marketing | Expanders - Apply equivalent subjects Search modes - Find all my search terms | Interface - EBSCOhost Research Databases Search Screen - Advanced Search Database - CINAHL Plus with Full Text | Display |
| S4 | TI food industry OR AB food industry | Expanders - Apply equivalent subjects Search modes - Find all my search terms | Interface - EBSCOhost Research Databases Search Screen - Advanced Search Database - CINAHL Plus with Full Text | Display |
| S3 | TI ( supermarkets or grocery stores or food retail ) OR AB ( supermarkets or grocery stores or food retail ) | Expanders - Apply equivalent subjects Search modes - Find all my search terms | Interface - EBSCOhost Research Databases Search Screen - Advanced Search Database - CINAHL Plus with Full Text | Display |
| S2 | TI obesogenic environment OR AB obesogenic environment | Expanders - Apply equivalent subjects Search modes - Find all my search terms | Interface - EBSCOhost Research Databases Search Screen - Advanced Search Database - CINAHL Plus with Full Text | Display |
| S1 | TI ( food environment or built food environment ) OR AB ( food environment or built food environment ) | Expanders - Apply equivalent subjects Search modes - Find all my search terms | Interface - EBSCOhost Research Databases Search Screen - Advanced Search Database - CINAHL Plus with Full Text | Display |

**WEB OF SCIENCE**

"food environment" OR "local food environment" OR "nutrition environment" OR "community environment" OR "consumer environment" OR "store environment" OR "obes* environment" OR "commercial determinants" OR "food marketing" OR "food industry" OR "obesity news" OR "food activism" or "online food" or "obesity policy" or "food policy" OR "fast foods" OR "Ultra processed food" OR "ultra-processed food" OR "processed food" OR "high in fat, salt and sugar" OR "junk food" OR living with obesity OR responsibility obesity OR discourse obesity

AND

gb OR "g.b." OR britain OR britisH OR uk OR "u.k." OR united kingdom* OR england OR english OR northern AND ireland OR northern AND irish* OR scotland* OR scottish* OR wales OR "south wales" OR welsh*

**GEOBASE**

found in Compendex, Inspec & GEOBASE for 1884-2024: ((("food environment" OR "local food environment" OR "nutrition environment" OR "community environment" OR "consumer environment" OR "store environment" OR "obes* environment" OR "commercial determinants" OR "food marketing" OR "food industry" OR "obesity news" OR "food activism" or "online food" or "obesity policy" or "food policy" OR "fast foods" OR "Ultra processed food" OR "ultra-processed food" OR "processed food" OR "high in fat, salt and sugar" OR "junk food" OR living with obesity OR responsibility obesity OR discourse obesity) WN KY) AND ((gb OR "g.b." OR britain OR britisH OR uk OR "u.k." OR united kingdom* OR england OR english OR northern AND ireland OR northern AND irish* OR scotland* OR scottish* OR wales OR "south wales" OR welsh*) WN KY))
