# Supplementary material for: Mapping how responsibility for poor diets is framed in the United Kingdom: a scoping review
Source: Public Health Nutr. 2025 Sep 22;28(1):e167. doi: 10.1017/S1368980025101079 (PMC12722098; doi:10.1017/S1368980025101079)
Supplement: Serrano-Fuentes et al. supplementary material 3 — Serrano-Fuentes et al. supplementary material [file S1368980025101079sup003.docx]

**Supplementary material 3. Deductive qualitative content analysis**

Deductive qualitative content analysis was used to guide the analysis ^(1)^. Content analysis was chosen as the analysis method in this scoping review because it is descriptive in nature, and the process of open coding to assign characteristics or concepts into overall categories can be applied to any study design or evidence source; it is not restricted to primary qualitative research ^(2)^. It was deductive because we operationalised it on the basis of previous knowledge ^(3)^. Thus, the initial coding was based on the research questions and an analytical framework developed, informed by multilevel health determinants theory ^(4–6)^, distinguishing between societal levels (micro-level, community-level, and structural-level factors). This helped systematically categorise the diverse range of influences on food choices identified across studies. The analysis had three phases:

1. Preparation: authors are immersed in the data, and an initial reading of all evidence sources is conducted so that they become familiar with the content.

2. Organising: an initial unconstrained framework was already decided on, and data began to be extracted according to it. In this pre-exisiting framework, categories were first reflected, followed by sub-categories and codes. There were 3 categories: i) public voice, ii) mass media and iii) UK government policies, and 2 sub-categories per each: i) individual responsibility and ii) environmental responsibility. The sub-category ‘environmental/societal responsibility’ was broken down in the codes ‘food industry’ and ‘UK Government’ since there was a particular interest in these two powerful institutions as part of the discourse (as theoretically justified in the background section of the review).

Version 1 of the framework

| Public voice | Individual responsibility  Environment/societal responsibility  Food industry  UK Government |
| --- | --- |
| Mass media | Individual responsibility  Environment/societal responsibility  Food industry  UK Government |
| UK Government | Individual responsibility  Environment/societal responsibility  Food industry  UK Government |

The framework was reviewed and ensured that the information addressed the review questions. Thus, the second version of the framework added pre-established codes concerning multilevel factors in order to organise all the environmental factors in a more structured way according to the multiple societal levels. Individual-level factors were interpreted as indicating individual responsibility attributions, while external factors (structural-level, community-level and micro-level) were interpreted as indicating societal responsibility attributions.

Version 2 of the framework

| Public voice | Individual-level factors (individual responsibility)  External factors (societal responsibility)  Structural-level factors  Food environment  Working demands  Pace of life  Social media  Food industry  Health system  Social norms  Policies  Economy  Community-level factors  Community social networks  Community assets  Micro-level factors  Close social networks |
| --- | --- |
| Mass media | Individual-level factors (individual responsibility)  External factors (societal responsibility)  Structural-level factors  Food environment  Working demands  Pace of life  Social media  Food industry  Health system  Social norms  Policies  Economy  Community-level factors  Community social networks  Community assets  Micro-level factors  Close social networks |
| UK Government | Individual-level factors (individual responsibility)  External factors (societal responsibility)  Structural-level factors  Food environment  Working demands  Pace of life  Social media  Food industry  Health system  Social norms  Policies  Economy  Community-level factors  Community social networks  Community assets  Micro-level factors  Close social networks |

The third and last versions of the framework were modified inductively since the initial versions did not provide the most accurate descriptive map of the available evidence. The frameworks were reviewed by the rest of the team members throughout the analysis process.

Version 3 of the framework

| Public voice | **- Genetic and biological factors**  **- Individual-level factors (individual responsibility)**  Self-control  Deficiencies in knowledge  Attitudes and behaviours  Lack of motivation  Willpower  Lack of cooking skills  Education  Role modelling  **- Individual responsibility (influenced by external factors)**   - Structural-level factors   Financial constraints  Restricted family budget  High costs of healthier food  Time scarcity  Childcare  Work schedules  Cultural norms  Food industry  Marketing strategies  UK government   - Community-level factors   Schools  Accessibility  No local shops with healthy food  No transport  Exposure to unhealthy food  Saturation of takeaways restaurants and convenience stores  Amount of unhealthy food available  Peer influence   - Micro-level factors   Family influences  Pressure from children and partners  Historical family influences  **- External factors (societal responsibility)**   - Structural-level factors   Financial constraints  Restricted family income  High costs of healthier food  Time scarcity  Childcare  Work schedule  Activities outside the home  Food industry  Marketing strategies  Media and advertising   - Community-level factors   Accessibility  No local shops with healthy food  No transport  Exposure to unhealthy food  Amount of unhealthy food available  Schools   - Micro-level factors   Family influences  Pressure from children  Historical family influences |
| --- | --- |
| Mass media | **- Genetic and biological factors**  **- Individual-level factors (individual responsibility)**  Self-control  Deficiencies in knowledge  Attitudes and behaviours  Education  Parents  **- External factors (societal responsibility)**   - Structural-level factors   Financial constraints  High costs of healthier food  Food industry  UK Government  Health authorities   - Community-level factors   Nature of living environments  Accessibility  Healthy food  Exposure to unhealthy food  Amount of unhealthy food available |
| UK Government | **- Individual-level factors (individual responsibility)**  Knowledge  Behaviours and lifestyles  Parents  **- External factors (societal responsibility)**   - Structural-level factors   Food industry  UK Government   - Community-level factors   Living environment (obesogenic) |

3. Reporting: the final phase was writing up, weaving together the analytic narrative and data extracts and contextualising the analysis regarding current literature.

References

1. Elo S & Kyngäs H (2008) The qualitative content analysis process. *J Adv Nurs* **62**, 107–115.

2. Pollock D, Peters MDJ, Khalil H, et al. (2023) Recommendations for the extraction, analysis, and presentation of results in scoping reviews. *JBI Evidence Synthesis* **21**, 520.

3. Burns N & Grove SK (2005) *The Practice of Nursing Research:Conduct, Critique & Utilization.* Elsevier Saunders. St Louis: .

4. Serrano Fuentes N (2023) Exploring the impact of multilevel environments influencing the adoption of health practices in adults with obesity in the United Kingdom. phd, University of Southampton.

5. Bronfenbrenner U (1979) *The Ecology of Human Development: Experiments by Nature and Design*. United States: Harvard University Press.

6. Dahlgren G & Whitehead M (1991) *Policies and strategies to promote social equity in health*. Stockholm, Sweden: Institute for Futures Studies.
